# RIT1 controls actin dynamics via complex formation with RAC1/CDC42 and PAK1

**DOI:** 10.1371/journal.pgen.1007370

**Published:** 2018-05-07

**Authors:** Uta Meyer zum Büschenfelde, Laura Isabel Brandenstein, Leonie von Elsner, Kristina Flato, Tess Holling, Martin Zenker, Georg Rosenberger, Kerstin Kutsche

**Affiliations:** 1 Institute of Human Genetics, University Medical Center Hamburg-Eppendorf, Hamburg, Germany; 2 Institute of Human Genetics, University Hospital Magdeburg, Magdeburg, Germany; Stanford University School of Medicine, UNITED STATES

## Abstract

RIT1 belongs to the RAS family of small GTPases. Germline and somatic *RIT1* mutations have been identified in Noonan syndrome (NS) and cancer, respectively. By using heterologous expression systems and purified recombinant proteins, we identified the p21-activated kinase 1 (PAK1) as novel direct effector of RIT1. We found RIT1 also to directly interact with the RHO GTPases CDC42 and RAC1, both of which are crucial regulators of actin dynamics upstream of PAK1. These interactions are independent of the guanine nucleotide bound to RIT1. Disease-causing *RIT1* mutations enhance protein-protein interaction between RIT1 and PAK1, CDC42 or RAC1 and uncouple complex formation from serum and growth factors. We show that the RIT1-PAK1 complex regulates cytoskeletal rearrangements as expression of wild-type RIT1 and its mutant forms resulted in dissolution of stress fibers and reduction of mature paxillin-containing focal adhesions in COS7 cells. This effect was prevented by co-expression of RIT1 with dominant-negative CDC42 or RAC1 and kinase-dead PAK1. By using a transwell migration assay, we show that RIT1 wildtype and the disease-associated variants enhance cell motility. Our work demonstrates a new function for RIT1 in controlling actin dynamics via acting in a signaling module containing PAK1 and RAC1/CDC42, and highlights defects in cell adhesion and migration as possible disease mechanism underlying NS.

## Author summary

Noonan syndrome (NS) belongs to the RASopathies, a group of developmental diseases caused by mutations in genes encoding RAS-MAPK pathway components. Germline mutations in *RIT1* have been identified in NS. RIT1 belongs to the RAS superfamily, however, the cellular function of RIT1 remains elusive. We show that RIT1 binds p21-activated kinase 1 (PAK1), an effector of the RHO GTPases RAC1 and CDC42, which are important regulators of cytoskeletal dynamics. NS-associated RIT1 mutants enhance complex formation between RIT1, RAC1/CDC42 and PAK1. Expression of wild-type or mutant forms of RIT1 caused loss of stress fibers and mature focal adhesions and enhanced cell motility. Our data suggest that dysfunction in actin dynamics is a novel aspect in the pathophysiology of RASopathies.

## Introduction

RIT1 belongs to the RAS superfamily of low molecular weight GTP-binding proteins that function as guanine nucleotide-regulated molecular switches in the cell by changing between an active GTP-bound and an inactive GDP-bound state [1, 2]. The RAS GTPases contain five well-conserved amino acid motifs, with G1 and G3 involved in phosphate binding, G2 in effector binding and G4 and G5 in GTP binding and hydrolysis [1]. Rit1 is expressed in a variety of tissues and throughout development [3]. A series of studies in primary neurons and pheochromocytoma (PC) cell lines indicated roles of Rit1 in neuronal morphogenesis [4, 5], neural differentiation [6–8], and cell survival [2, 8–10]. Detailed biochemical analyses showed that Rit1 mediates cell survival via the p38-MK2-HSP27 and mTORC2-Akt signaling pathways [9–12] and regulates MEK-ERK signal transduction upon activation of particular upstream cell surface receptors in certain cell types [6, 8, 13].

**Fig 1:**
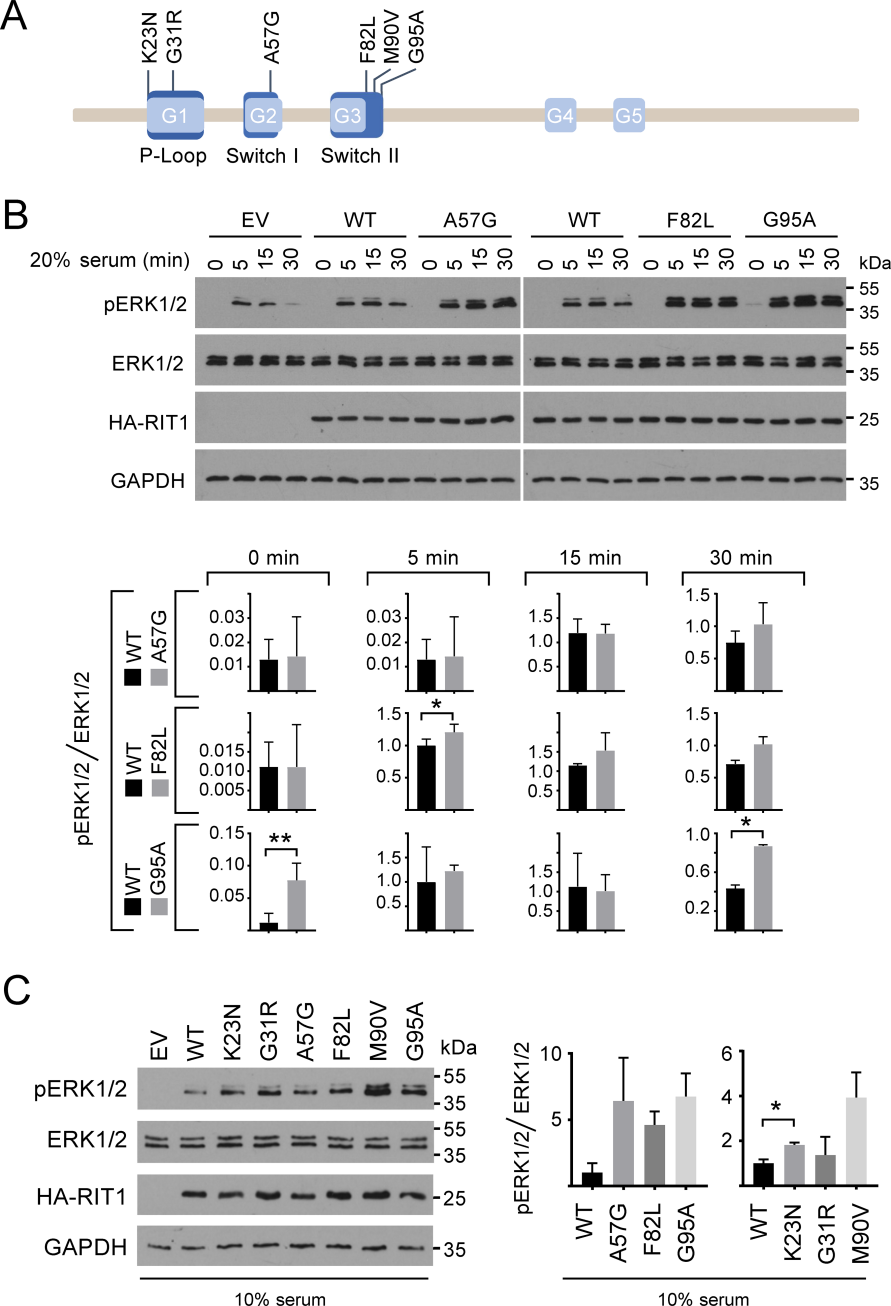
Expression of RIT1 mutants enhances phosphorylation of ERK1/2. (A) Schematic representation of RIT1 with selected NS-associated amino acid substitutions. RIT1 isoform 2 (protein RefSeq NP_008843.1) comprises 219 amino acids and has five conserved GDP/GTP binding motifs (G1 to G5, light blue). Motifs representing the P-loop and switch I and II regions are shown in dark blue. The P-loop binds γ-phosphate of GTP and GDP, the switch regions are critical for GDP/GTP binding and for interaction with upstream and downstream partners. RIT1 amino acid substitutions identified in patients with NS and selected for functional studies in this work are given in the one-letter code above the scheme. (B) HEK293T cells were transfected with empty vector (EV) or constructs expressing HA-RIT wildtype (WT), HA-RIT1 p.A57G, p.F82L, or p.G95A as indicated. Cells were serum-starved (0.1% serum) and subsequently stimulated with 20% serum for 5, 15, or 30 min or left untreated (0 min). Cell extracts were analyzed by immunoblotting using anti-phospho-ERK1/2 (pERK1/2) and anti-ERK1/2 (ERK1/2) antibodies. Expression of HA-tagged RIT1 protein variants was monitored by immunoblotting using anti-HA antibody, and anti-GAPDH antibody was used to control for equal loading. Data shown are representative of three independent experiments. Autoradiographic signals were quantified by scanning densitometry. Levels of phosphorylated ERK1/2 were normalized relative to amounts of total ERK1/2. To conserve the relative variance of the samples, values for RIT1 wildtype and mutants were divided by the mean of the wildtype samples [79]. Graphs show phosphorylation levels upon serum starvation (0 min) and after 5, 15, and 30 min serum stimulation in cells expressing RIT1 wildtype (WT), RIT1 p.A57G, p.F82L or p.G95A (arbitrary units). The mean of three independent experiments ± SD is given. Unpaired *t*-tests were used to determine statistical significance (*, *P* < 0.05; **, *P* < 0.01). (C) HEK293T cells were transfected with empty vector (EV) and RIT1 expression constructs (WT, p.K23N, p.G31R, p.A57G, p.F82L, p.M90V, and p.G95A) as indicated and cultured under steady-state condition (10% serum). Total cell lysates were analyzed as described in (B). Levels of phosphorylated ERK1/2 were normalized relative to amounts of total ERK1/2. To conserve the relative variance of the samples, values for RIT1 wildtype and mutants were divided by the mean of the wildtype samples [79]. The graph on the left shows relative phosphorylation levels in cells expressing RIT1 wildtype (WT), RIT1 p.A57G, p.F82L or p.G95A; the graph on the right shows relative phosphorylation levels in cells expressing RIT1 wildtype (WT), RIT1 p.K23N, p.G31R or p.M90V (arbitrary units). The mean of three independent experiments ± SD is given. Statistical significance was assessed by one-way ANOVA: not significant and *P* < 0.01 for RIT1 variants shown in the left and right graph, respectively. *Post hoc P* values were calculated by *t*-tests and Bonferroni correction; *, *P* < 0.05.

The recent identification of germline gain-of-function *RIT1* mutations in patients with Noonan syndrome (NS) demonstrated the importance of this small GTPase for embryonic development [14]. NS is a genetically heterogeneous, autosomal dominant disorder characterized by craniofacial dysmorphism, growth retardation, cardiac abnormalities, and learning difficulties [15]. Individuals with a *RIT1* mutation have a higher prevalence of cardiovascular manifestations and lymphatic problems compared with other NS subtypes, and the frequency of *RIT1* mutations in NS is at least 5% [14, 16–18]. NS-associated *RIT1* mutations reported to date particularly affect codons 57, 82 and 95 and result in amino acid changes in the switch I and II regions (Fig 1A) [14, 16–30]. These two protein motifs are involved in nucleotide, effector and regulator binding and thereby ensure molecular functioning of RIT1 [31, 32]. Almost all causative genes for NS and clinically overlapping diseases encode components or regulators of RAS-mediated signaling, and dysregulated RAS-MAPK signaling has been postulated to be the pathogenic mechanism shared among these disorders, summarized as RASopathies [33–35].

Rit1 shares effector molecules with Ras, such as Raf1 and the p110 catalytic subunit of PI3K [2, 36]. Accordingly, EGF-induced ERK activation has previously been found to be enhanced and/or sustained in T-REx293T cells expressing NS-associated RIT1 mutants [21], and ectopic expression of NS- and cancer-related RIT1 mutants in PC6 cells induced phosphorylation of both MEK and ERK [25, 37]. Similarly, the NS-associated RIT1 mutants p.A77S and p.M90I, also found in lung adenocarcinoma, stimulated phosphorylation of AKT in PC6 cells [37]. Collectively, the data suggest that RIT1 is a positive modulator of RAF-MEK-ERK and PIK3-AKT signaling, and NS-associated mutants cause sustained activation of both pathways. With the identification of *RRAS* as RASopathy-associated gene [38], that encodes a small GTPase controlling cell adhesion, spreading and migration [39–41], the question arose whether signaling routes other than RAF-MEK-ERK and PIK3-AKT may also be relevant in the pathogenesis of RASopathies. In line with this, expression of the constitutively active Rit1 mutant Q79L changes NIH 3T3 cellular morphology, characterized by membrane extensions with some ruffle-like structures at their end [42]. Induction of these actin-dependent structures by active Rit1 suggests an involvement of this small GTPase in cytoskeletal rearrangements. In general, the Rho GTPases Rho, Rac and Cdc42 act as key regulators of actin dynamics and associated processes, such as cell migration [43]. Indeed, Rac/Cdc42 was found in a ternary complex with Rit1 and the cell-polarity protein Par6 [44, 45].

Here we provide evidence for a novel signaling node containing RIT1 and major cytoskeleton regulators and emphasize altered actin dynamics as another aspect in the molecular pathogenesis of NS.

## Results

### NS-associated RIT1 mutants increase ERK1/2 phosphorylation

We studied the signaling consequences of wild-type RIT1 (WT) and six NS-associated *RIT1* mutations, including p.K23N and p.G31R in the P-loop, p.A57G in the switch I region, and p.F82L, p.M90V and p.G95A in the switch II region (Fig 1A) [14, 16–18, 27]. Serum stimulation promoted a sound ERK1/2 phosphorylation response in HEK293T cells expressing wild-type RIT1, which was increased compared to control cells (Fig 1B), indicating that RIT1 affects MEK-ERK signaling. All six RIT1 mutants induced elevated and prolonged phosphorylation of ERK1/2 upon serum stimulation compared with RIT1 WT (Fig 1B and S1A Fig). This increase was statistically significant for RIT1 p.F82L (5 min), p.G95A (30 min) and p.M90V (5, 15 and 30 min) (Fig 1B and S1A Fig). Only RIT1 p.G95A was able to (statistically significantly) stimulate ERK1/2 phosphorylation under serum-deprived culture condition suggesting a growth factor-independent function of this mutant (Fig 1B and S1B Fig). Under basal, steady-state condition (10% serum), RIT1 wildtype expression resulted in a moderate activation of ERK1/2 that was elevated in cells expressing any of the RIT1 mutants (Fig 1C). *Post hoc* testing revealed statistical significance for the RIT1 mutant p.K23N (Fig 1C). A minor effect of the p.G31R mutant on ERK1/2 activation was confirmed under steady-state condition (Fig 1C and S1C Fig). Together, these data confirm a role of RIT1 in ERK activation and demonstrate that NS-associated RIT1 mutants intensify ERK1/2 phosphorylation.

Next, we determined phosphorylation levels of AKT at both serine 473 and threonine 308 in HEK293T cells expressing RIT1 wildtype and three of the mutants. Phosphorylation of serine 473 was similar in all analyzed cell lysates, and even stimulation with serum did not induce AKT phosphorylation at this residue (S2A Fig). Phosphorylation of threonine 308 could be slightly stimulated by EGF, however, there was no difference in AKT phosphorylation between control and RIT1 wildtype expressing cells (S2B Fig). These data indicate that AKT signaling is very robust in HEK293T cells, and responsiveness to serum factors is limited.

### RIT1 interaction with PAK1 is stimulated by serum factors

**Fig 2:**
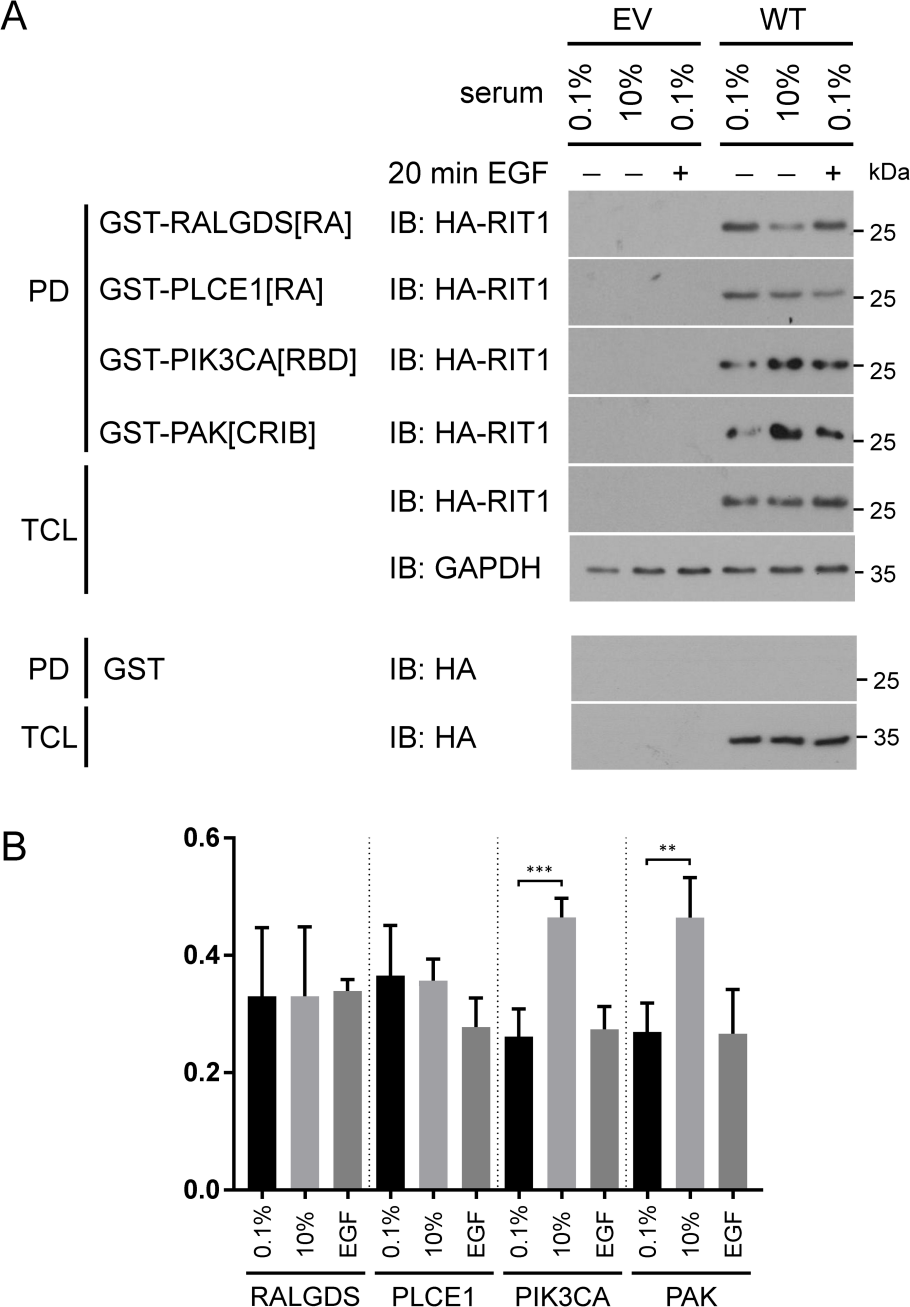
Serum and EGF stimulate the association of RIT1 with PIK3CA and PAK1. (A) HEK293T cells expressing empty vector (EV) or wild-type RIT1 (WT) were either serum-deprived (0.1%) and subsequently stimulated with 10 ng/ml EGF for 20 min (0.1%; +) or kept under full serum (10%). GTP-bound HA-tagged RIT1 was precipitated from cell extracts using GST-RALGDS[RA], GST-PLCE1[RA], GST-PIK3CA[RBD], and GST-PAK[CRIB] fusion proteins (PD, pull down). Precipitated HA-RIT1 (PD) and HA-RIT1 in the total cell lysates (TCL) were detected by immunoblotting (IB) using anti-HA antibody. Cellular extracts were probed with anti-GAPDH antibody to control for equal loading. For control purpose cell extracts were incubated with GST-coupled agarose beads. Data shown are representative of three independent experiments. (B) The graph shows the relative amount (arbitrary units) of co-precipitated RIT1 protein. The mean of four independent experiments ± SD is given. One-way ANOVA between groups: *P* < 0.01. *Post hoc P* values were calculated by *t*-tests and Bonferroni correction; **, *P* < 0.01; ***, *P* < 0.001.

To identify novel effector molecules of RIT1 and study their biological relevance, we performed binding assays under different culture conditions (0.1% serum, 10% serum basal condition, and 0.1% serum followed by 20 min EGF stimulation). We first tested three known RAS effectors and could pull down RIT1 with GST-RALGDS[RA], GST-PLCE1[RA] and GST-PIK3CA[RBD] (as positive control) under all tested cell culture conditions (Fig 2A). As a negative control, we used GST alone (Fig 2A). The amount of HA-RIT1 co-precipitated with GST-RALGDS[RA] or GST-PLCE1[RA] was the same or even less in the presence of serum or upon EGF stimulation compared with serum-starved condition (0.1% serum) (Fig 2A and 2B). In contrast, we observed a statistically significant increase of HA-RIT1 in PIK3CA::RBD precipitates from cells cultured in the presence of serum factors compared to serum-starved cells (Fig 2A and 2B). Together, the data suggest that RALGDS and PLCE1 are binding to RIT1, however, the interaction is not stimulated by growth or serum factors. PIK3CA seems to be a physiological relevant RIT1 binding partner, as has been demonstrated by others [37, 46].

Rit1 has been found in a protein complex with Rac/Cdc42 and Par6 [44, 45]. p21-activated kinases (PAKs) act downstream of Rho family members and are critical for multiple signaling pathways associated with cell growth, cytoskeletal dynamics, cell polarity, survival, and development [47, 48]. We tested if RIT1 is part of a PAK-containing protein complex and used the GST-tagged Rac/Cdc42 interactive binding (CRIB) domain of PAK1 (PAK[CRIB]) in pull down assays. Indeed, HA-RIT1 co-precipitated with PAK[CRIB] under all culture conditions tested (Fig 2A). We detected a significantly increased amount of RIT1 in precipitates from cells cultured in the presence of serum factors (10% serum) compared to serum-starved (0.1% serum) cells (Fig 2B). This data suggests physiological relevance of a RIT1- and PAK1-containing protein complex in HEK293T cells.

### RIT1 and PAK1 directly interact, and *RIT1* germline mutations enhance formation of the complex

**Fig 3:**
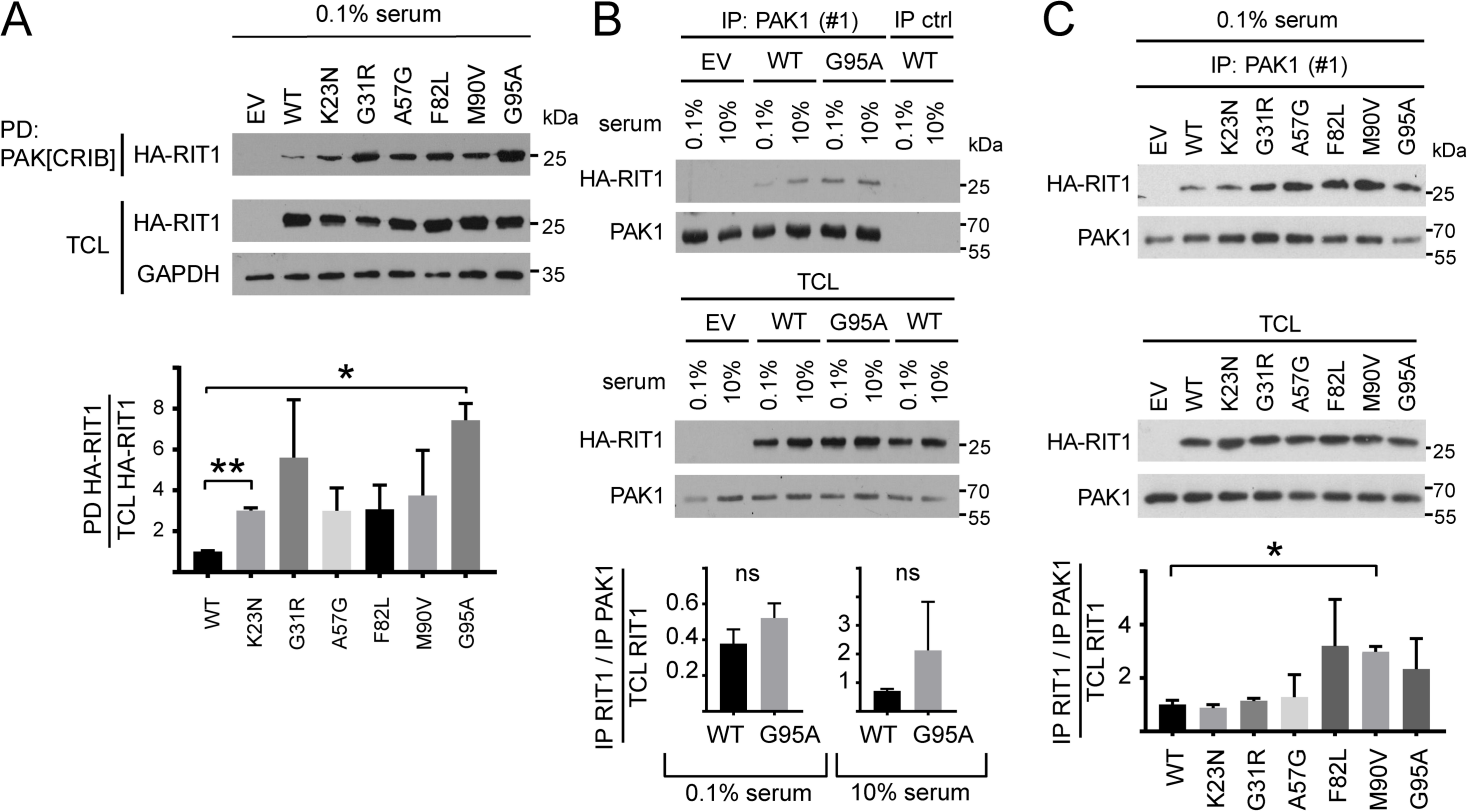
NS-associated RIT1 amino acid changes enhance binding of RIT1 to PAK1. (A) HEK293T cells were transfected with empty vector (EV) and RIT1 expression constructs (WT, p.K23N, p.G31R, p.A57G, p.F82L, p.M90V, and p.G95A) as indicated and cultured under serum-starved conditions (0.1% serum). HA-tagged RIT1 protein variants were precipitated from cell extracts using GST-PAK[CRIB] fusion proteins (PD, pull down). Precipitated HA-RIT1 (PD) and HA-RIT1 in the total cell lysates (TCL) were detected by immunoblotting using anti-HA antibody. Anti-GAPDH antibody was used to control for equal loading (TCL, total cell lysate). Data shown are representative of three independent experiments. Autoradiographic signals were quantified by scanning densitometry. The amount of co-precipitated HA-RIT1 was normalized relative to the amount of total HA-RIT1. To conserve the relative variance of the samples, values for RIT1 wildtype and mutants were divided by the mean of the wildtype samples [79]. The graphs show the relative amounts of co-precipitated RIT1 protein variants (arbitrary units). Data represent the mean of three independent experiments ± SD. One-way ANOVA between groups: *P* < 0.01. *Post hoc P* values were calculated by *t*-tests and Bonferroni correction; *, *P* < 0.05; **, *P* < 0.01. (B) HEK293T cells transfected with empty vector (EV) or expressing wild-type RIT1 (WT) or RIT1 p.G95A were either serum-deprived (0.1%) or kept under full serum (10%). Endogenous PAK1 was immunoprecipitated from cell extracts using an anti-PAK1 antibody [IP: PAK1 (#1)]. As IP control an irrelevant isotype-matched antibody (anti-pSMAD2 antibody) was used (IP ctrl). Co-precipitated HA-RIT1 and expression of HA-RIT1 in total cell lysates (TCL) was detected by immunoblotting using anti-HA antibody. Expression of endogenous PAK1 in TCL is shown below. Data shown are representative of three independent experiments. Autoradiographic signals were quantified by scanning densitometry. Levels of co-IPed HA-RIT1 were double-normalized relative to amounts of immunoprecipitated PAK1 and HA-RIT1 in total cell lysates. To conserve the relative variance of the samples, values for RIT1 wildtype and p.G95A were divided by the mean of the wildtype samples [79]. The graphs show the relative amount of co-precipitated HA-RIT1 in cells expressing RIT1 WT and RIT1 p.G95A (arbitrary units) and cultivated in 0.1% or 10% serum. The mean of three independent experiments ± SD is given. Unpaired *t*-tests were used to determine statistical significance. ns, not significant. (C) HEK293T cells were transfected with empty vector (EV) and RIT1 expression constructs as indicated and cultured under serum deprivation (0.1% serum). Endogenous PAK1 was immunoprecipitated with an anti-PAK1 antibody [IP: PAK1 (#1)], and co-precipitated HA-RIT1 was detected using an anti-HA antibody. Enrichment of PAK1 in the precipitates was demonstrated with an anti-PAK1 antibody. The amount of HA-RIT1 and PAK1 in TCL is shown. Data shown are representative of two independent experiments. Autoradiographic signals were quantified by scanning densitometry. Levels of co-IPed HA-RIT1 were double-normalized relative to amounts of immunoprecipitated PAK1 and HA-RIT1 in total cell lysates. To conserve the relative variance of the samples, values for RIT1 wildtype and RIT1 mutants were divided by the mean of the wildtype samples [79]. The graphs show the relative amount (arbitrary units) of co-precipitated RIT1 protein variants. The mean of two independent experiments ± SD is given. Unpaired *t*-tests were used to determine statistical significance (*, *P* <0.05).

Next, we aimed to investigate if NS-associated *RIT1* mutations affect formation of the novel RIT1-PAK1 complex. We pulled down HA-tagged RIT1 wildtype or mutants from HEK293T lysates by using PAK[CRIB]. Compared with wild-type RIT1, RIT1^K23N^ and RIT1^G95A^ significantly increased the amount of co-precipitated RIT1 (Fig 3A). Enhanced co-precipitation of RIT1^G31R^, RIT1^A57G^, RIT1^F82L^ and RIT1^M90V^ with PAK[CRIB] was detected in every single experiment, however, statistical significance was not reached (Fig 3A). We independently confirmed our findings by co-immunoprecipitation (co-IP) experiments. Both HA-RIT1 wildtype and HA-RIT1^G95A^ co-IPed with endogenous PAK1 from lysates of serum-deprived cells (0.1% serum) and from cells cultured in 10% serum (Fig 3B). In serum-deprived cells, co-precipitation of HA-RIT1^F82L^, HA-RIT1^M90V^ and RIT1^G95A^ with PAK1 tended to be stronger than of RIT1 wildtype; co-IPed HA-RIT1^M90V^ was significantly increased (Fig 3C). The trend of *RIT1* mutations to increase the amount of co-precipitated RIT1 with PAK1 also turned out by using a second anti-PAK1 antibody (S3A Fig). Under basal, steady-state condition (10% serum), binding of RIT1 wildtype and mutants to endogenous PAK1 was observed, but no difference in the amount of co-precipitated RIT1 wildtype or mutants was detected (S3B Fig). Interaction between endogenous PAK1 and RIT1 wildtype and the p.G95A mutant was also shown in COS7 cells (S4A–S4C Fig).

**Fig 4:**
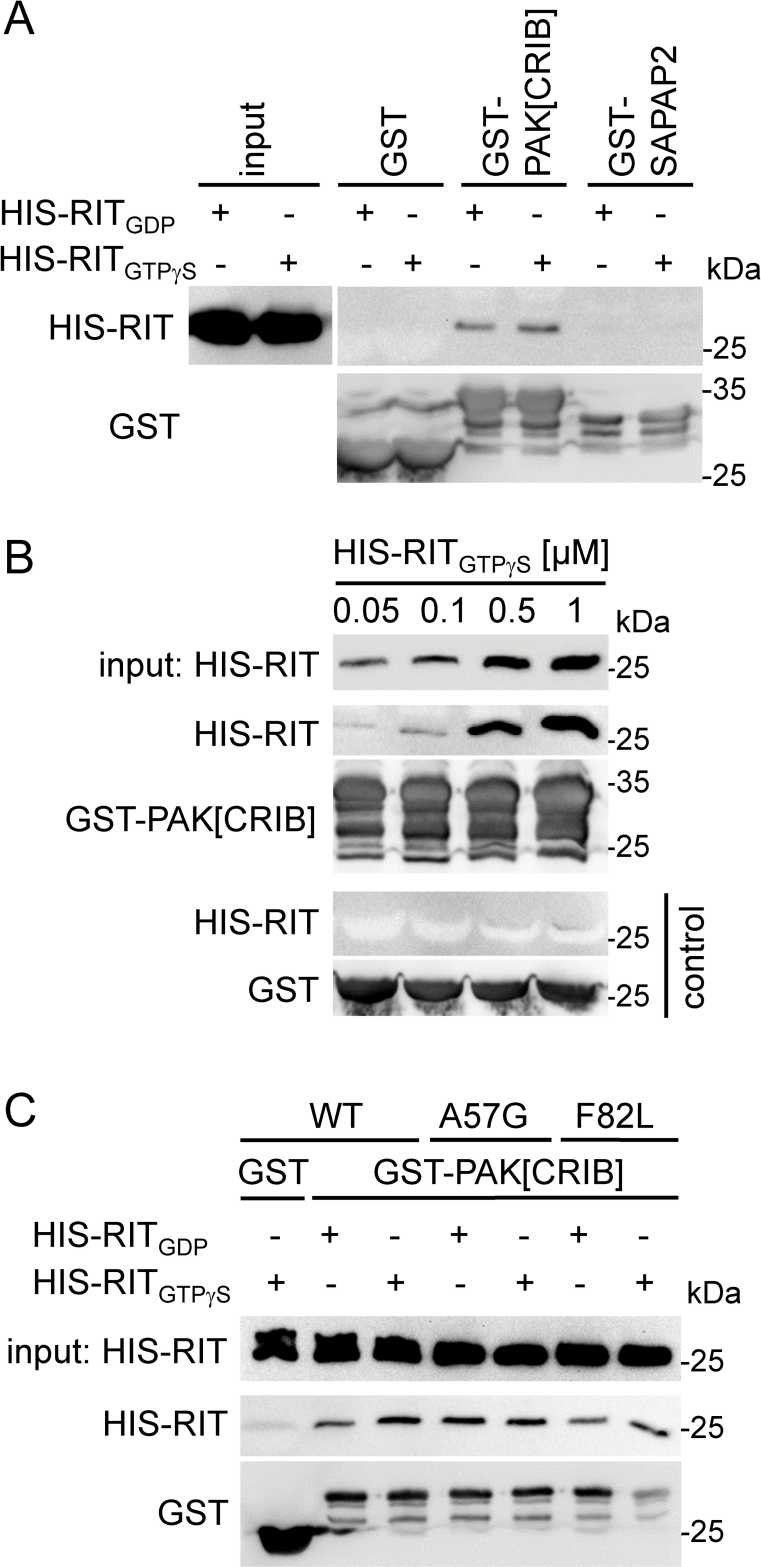
RIT1 directly interacts with PAK1. Recombinant His-tagged RIT1 wildtype (WT), p.A57G and p.F82L proteins (0.5 μM each) were loaded with GDP or non-hydrolyzable GTPγS as indicated, incubated with 1 μM GST-PAK[CRIB] bound to glutathione agarose, and precipitated. Samples were analyzed by immunoblotting using an anti-His antibody (precipitates and input) and an anti-GST antibody (precipitates). (A) RIT1 binds to PAK[CRIB]. To confirm specificity, recombinant GST-SAPAP2 (750 nM) and GST (1 μM) were used. (B) RIT1_GTPγS_ interacts with PAK[CRIB] in the nanomolar range. Final concentrations of His-RIT1_GTPγS_ are indicated in μM. As control, His-RIT1_GTPγS_ was incubated with GST alone (lower panels). (C) Both GDP- and GTPγS-loaded RIT1 mutants p.A57G and p.F82L interact with PAK[CRIB]. Data shown are representative of three (A and B) and two (C) independent experiments.

As PAKs fall into two categories, group I and group II, and PAK1 belongs to group I [48], we tested a member of group II, PAK4, for RIT1 binding. Neither wild-type HA-RIT1 nor the p.G95A mutant was detected in GFP-PAK4 precipitates (S4D Fig), indicating that preferentially group I PAKs form complexes with RIT1. By using an *in-vitro* binding assay, we detected a direct interaction of His-tagged RIT1 with GST-PAK[CRIB] that was slightly increased when RIT1 was in the GTPγS-bound state compared with the GDP-bound state (Fig 4A). RIT1 interacts with PAK[CRIB] in the nanomolar range indicating a medium binding affinity (Fig 4B). The switch I and II regions of RIT1 are involved in nucleotide, effector and regulator binding [31, 32]. Therefore, we analyzed the two mutants His-RIT1^A57G^ and His-RIT1^F82L^ affecting switch I and II, respectively (Fig 1A), in the *in-vitro* binding assay. Both showed a direct binding to PAK[CRIB] (Fig 4C). Taken together, our data demonstrate that RIT1 directly interacts with PAK1, and NS-associated *RIT1* mutations enhance the protein-protein interaction and uncouple it from serum factors. The strongest effects were seen for mutations located in the switch II region.

### RIT1 binds the RHO GTPases CDC42 and RAC1

**Fig 5:**
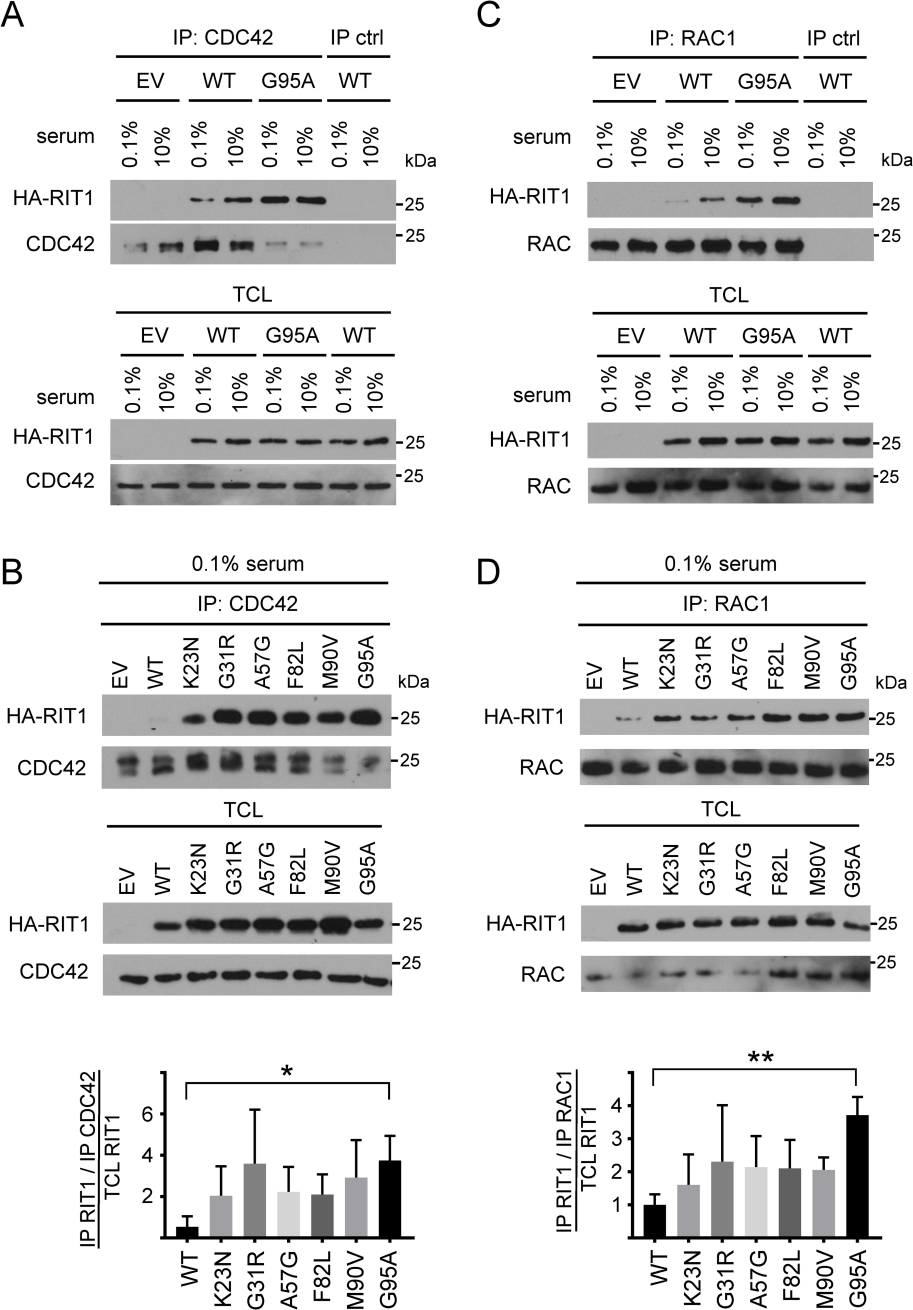
CDC42 and RAC1 interact with RIT1, and NS-associated RIT1 amino acid substitutions increase complex formation between RIT1 and RAC1/CDC42. (A and C) HEK293T cells transfected with empty vector (EV) or expressing wild-type RIT1 (WT) or RIT1 p.G95A were either serum-deprived (0.1% serum) or kept under full serum (10% serum). Endogenous CDC42 and RAC1 were immunoprecipitated with an anti-CDC42 antibody ([CDC42 (#1)] (A) and an anti-RAC1 antibody (C), respectively (IP). Co-precipitated HA-RIT1 and HA-RIT in total cell lysates (TCL) was detected by an anti-HA antibody. Enrichment of CDC42 and RAC1 in the precipitates and amount of endogenous CDC42 and RAC1 in TCL was demonstrated with an anti-CDC42 antibody ([CDC42 (#2)] (A) and an anti-RAC1/2/3 antibody (C), respectively. As a control, immunoprecipitation with an anti-V5 antibody was carried out (IP ctrl). Data shown are representative of three independent experiments. (B and D) HEK293T cells were transfected with empty vector (EV) and RIT1 expression constructs as indicated and cultured under serum deprivation (0.1% serum). Cell lysates were processed as described in (A) and (C). Data shown for CDC42 and RAC1 are representative of seven (B) and four (D) independent experiments, respectively. Autoradiographic signals were quantified by scanning densitometry. The amount of co-precipitated HA-RIT1 was double-normalized relative to amounts of immunoprecipitated CDC42/RAC1 and HA-RIT1 in total cell lysates. To conserve the relative variance of the samples, values for RIT1 wildtype and RIT1 mutants were divided by the mean of the wildtype samples [79]. The graphs show the relative amount (in arbitrary units) of co-precipitated RIT1 protein variants. The mean of five (B) and four (D) independent experiments ± SD is given. One-way ANOVA between groups: *P* < 0.05 (B and D). *Post hoc P* values were calculated by *t*-tests and Bonferroni correction; *, *P* < 0.05; **, *P* < 0.01 (B and D).

As RIT1 and PAK1 are in a protein complex and PAKs are prominent effectors of CDC42 and RAC1 [48], we tested for an association between RIT1 and CDC42 or RAC1. HA-RIT1 wildtype co-IPed with endogenous CDC42 from lysates of serum-deprived HEK293T cells (0.1% serum) and more efficiently from cells cultured in 10% serum (Fig 5A). The p.G95A mutation uncoupled enhanced complex formation between RIT1 and CDC42 from serum factors (Fig 5A). By performing further CDC42 co-IPs, we found RIT1 protein enriched in the precipitates from serum-deprived cells expressing either of the RIT1 mutants compared to RIT1 wildtype (Fig 5B). *Post hoc* testing showed statistically significant enrichment for the RIT1 p.G95A mutant compared with wildtype (Fig 5B). Under basal, steady-state condition (10% serum), the amount of RIT1 wildtype and each of the mutants that co-IPed with CDC42 was not significantly different (S5A Fig).

In an analogous set of experiments, we immunoprecipitated endogenous RAC1 and detected RIT1 wildtype in the precipitate; the amount of RIT1 p.G95A was increased in the RAC1 precipitates under both culture conditions (0.1% and 10% serum; Fig 5C). Similarly, each of the six *RIT1* mutations enhanced co-immunoprecipitation of RIT1 with RAC1 in serum-deprived cells (Fig 5D). *Post hoc* testing revealed a significant enhanced amount of co-IPed RIT1 p.G95A (Fig 5D). In cells cultured under basal condition, the amount of co-IPed RIT1 was similar for RIT1 wildtype and mutants (S5B Fig). We confirmed association of RAC1 or CDC42 with RIT1 by co-IPs and myc-trap assays in HEK293T cells. Wild-type RIT1 co-IPed with ectopically expressed RAC1 or CDC42, and the p.G95A substitution strengthened co-immunoprecipitation efficiency (S6A and S6B Fig). To demonstrate specificity of RAC1 and CDC42 binding to RIT1, we used RHOA, another member of the RHO family of GTPases, in GFP-trap assays; neither RIT1 wildtype nor the p.G95A mutant was found in the precipitates of ectopically expressed EGFP-tagged RHOA (S6C Fig). By using the Flp-In system that allows integration of the gene of interest at a specific genomic location, we generated isogenic Flp-In 293 cell lines stably and moderately expressing the RIT1 mutant p.G31R or p.A57G. We used these cell lines, which fairly reflect disease relevant conditions, to demonstrate interaction of endogenous CDC42 with RIT1 p.G31R and p.A57G. Both HA-tagged RIT1 p.G31R and p.A57G co-immunoprecipitated with CDC42 (S6D Fig). Finally, by *in-vitro* binding assays, we tested if interaction between RIT1 and RAC1 or CDC42 is direct. RIT1 and CDC42 as well as RIT1 and RAC1 show a direct binding, and both of which seemed to be independent of the bound guanine nucleotide (GDP vs. GTPγS) (S7A and S7B Fig). Taken together, our data demonstrate that RIT1 can directly bind to PAK1 and also to RAC1 and CDC42, suggesting the existence of a multiprotein signaling module.

### Wild-type RIT1 and NS-associated RIT1 mutants induce dissolution of stress fibers that is mediated by PAK1

**Fig 6:**
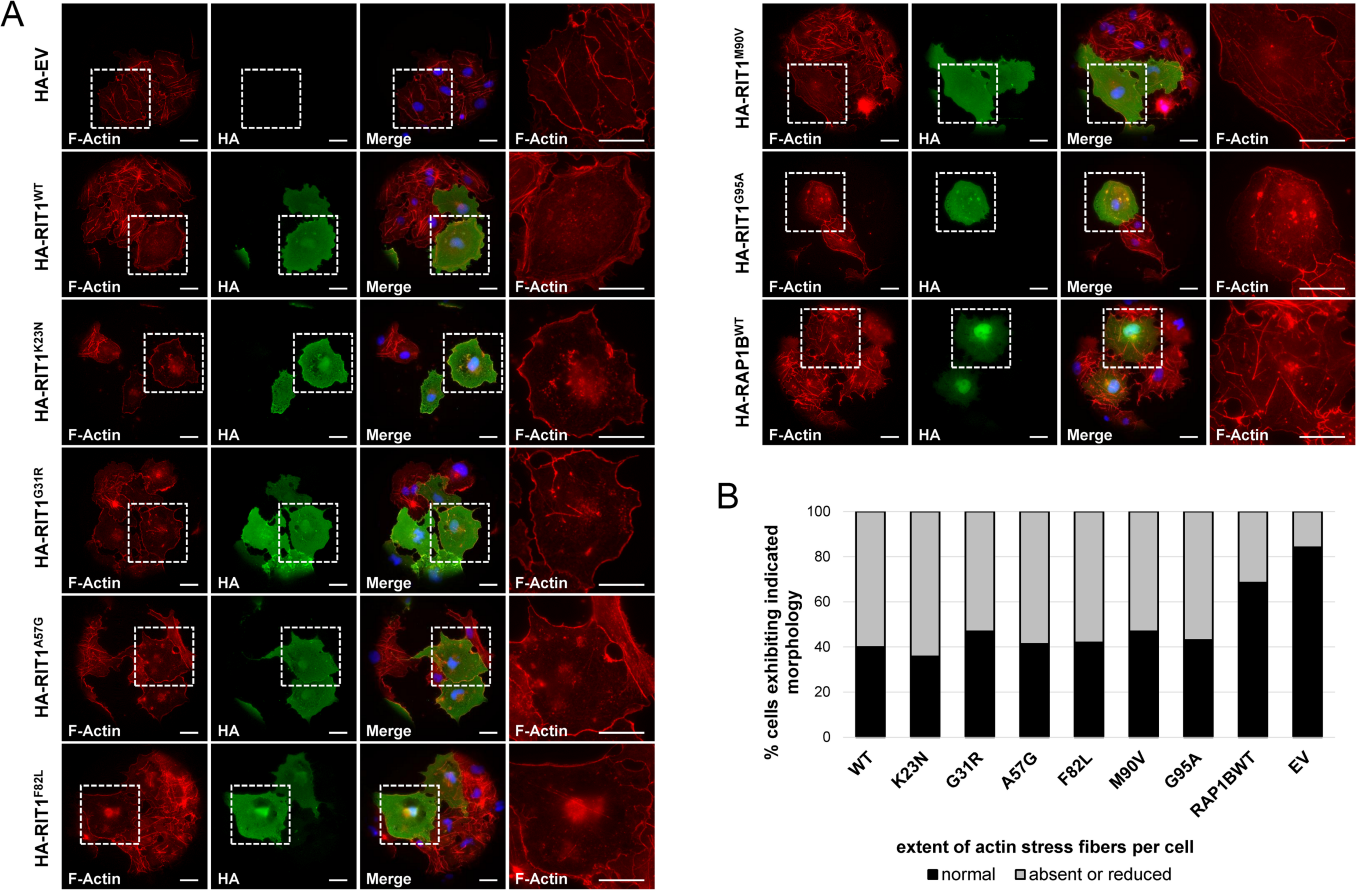
Dissolution of actin stress fibers in cells expressing RIT1 and NS-associated RIT1 mutants. (A) COS7 cells were plated on collagen-coated glass slides, transiently transfected with the indicated constructs and kept under serum starvation overnight. HA-tagged RIT1 was stained by rabbit anti-HA antibody followed by anti-rabbit Alexa Fluor488-conjugated antibody. Polymerized F-actin was visualized using Texas Red-X Phalloidin, and nuclear DNA was labeled by DAPI. Cells were imaged by epifluorescence microscopy. White boxes indicate magnified parts of specimen shown on the very right-hand side. Scale bars, 10 μm. (B) Cells were divided into the two indicated categories: (I) normal and (II) reduced or absent actin stress fibers (S8B Fig shows exemplary images). A minimum of 50 cells per dataset were analyzed.

The major function of RAC1, CDC42, and PAK1 is the regulation of the actin cytoskeleton to modulate cell shape, motility and adhesion [48–50]. Active Pak1 promoted the loss of actin stress fibers [51, 52]. Stress fibers are contractile actin-myosin filaments that are directly linked to focal adhesions which provide the main sites of cell adhesion to extracellular matrix [53]. To investigate a possible role of RIT1 in stress fiber and focal adhesion regulation, we utilized COS7 cells because HEK cells were loosely adherent and exhibited a poorly organized actin cytoskeleton (S8A Fig) [54, 55]. While cells transfected with empty vector possessed well-organized, prominent bundles of actin stress fibers throughout the cell bodies, RIT1-expressing cells showed a drastic loss of stress fibers from the cell interior (Fig 6A). The same effect, i.e. dissolution of internal stress fibers was detected in cells expressing any of the six NS-associated RIT1 mutants (Fig 6A). To exclude that the morphological changes were due to protein overexpression in COS7 cells, we transiently expressed RAP1B, another small GTPase of the RAS family, and observed the same distribution and morphology of stress fibers as in cells transfected with empty vector (Fig 6A). To quantify the data, we determined the number of cells with absent or reduced stress fibers (S8B Fig). In COS7 cells expressing RIT1 wildtype or any of the mutants, 53–64% of cells showed no or a reduced number of stress fibers (Fig 6B). In contrast, only 31% and 16% of cells expressing RAP1B and transfected with empty vector, respectively, showed a reduced number or no stress fibers (Fig 6B).

It has been reported that loss of stress fibers promoted by Pak1 was accompanied by disappearance of focal adhesions [51, 52], and Pak-induced phosphorylation of the focal adhesion-specific protein paxillin increased adhesion turnover [56, 57]. Thus, we studied the effect of RIT1 wildtype and mutants on the size and shape of paxillin-containing focal adhesions in COS7 cells. By quantification of both nascent (dot-like) and mature (stripe-like) focal adhesions, we observed a shift from mature to nascent focal adhesions: in COS7 cells expressing RIT1 wildtype or mutants, 71–74% of focal adhesions were nascent, while 58% and 56% of focal adhesions in RAP1B expressing cells and those transfected with vector, respectively, were nascent (S9A and S9B Fig). The data suggest that expression of RIT1, either wildtype or disease-associated mutants negatively affects the stability of stress fibers and maturation of focal adhesions.

**Fig 7:**
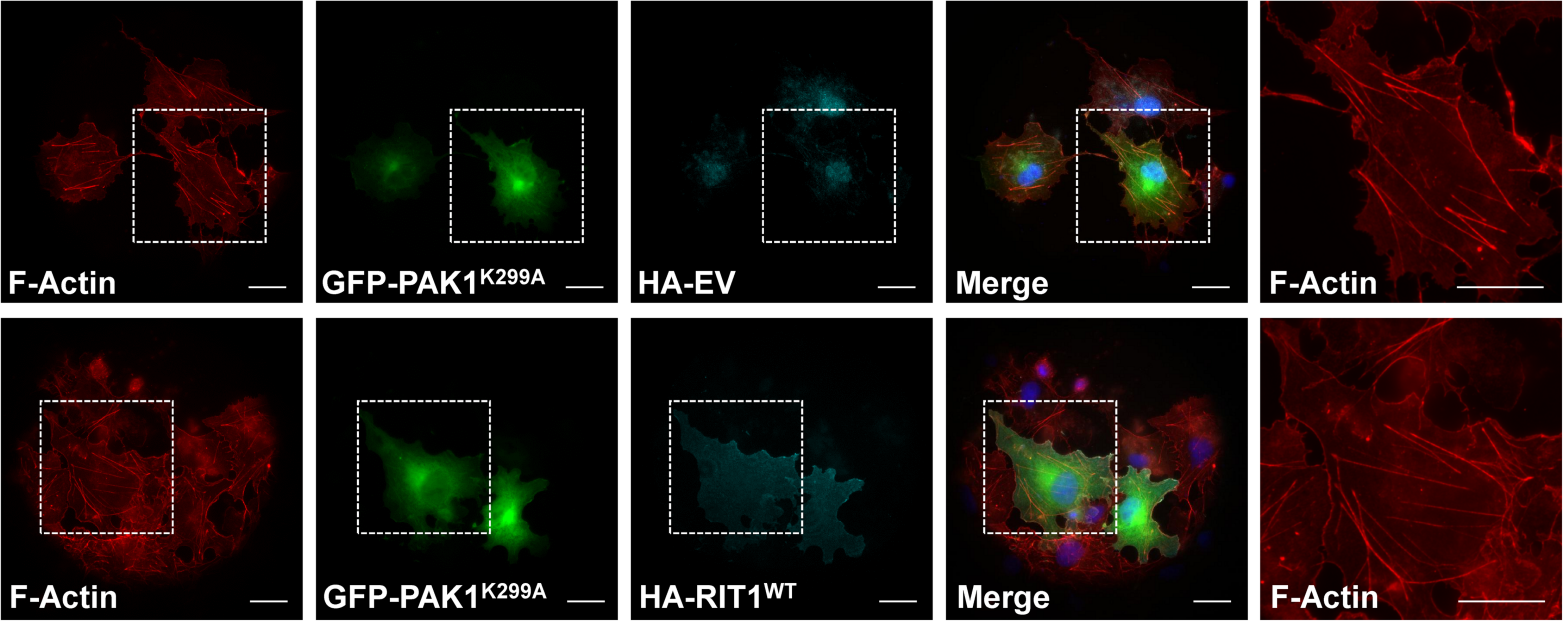
Co-expression of kinase-dead PAK1^K299A^ prevents loss of actin stress fibers in cells expressing RIT1. COS7 cells were plated on collagen-coated glass slides, transiently transfected with GFP-PAK1^K299A^ expression construct together with HA-RIT^WT^ construct or empty vector (EV) and serum-starved overnight. HA-tagged RIT1 was stained by rabbit anti-HA antibody followed by anti-rabbit Alexa Fluor647-conjugated antibody. F-actin was visualized using Texas Red-X Phalloidin, and nuclear DNA was labeled by DAPI. White boxes indicate magnified parts of specimen shown on the very right-hand side. Scale bar, 10 μm.

Expression of constitutively active forms of RAC1 and CDC42 (Q61L) also was associated with loss of stress fibers, and parallel blocking of PAK1’s activity prevented stress fiber dissolution [51, 58]. We analyzed the consequences of dominant-negative PAK^K299A^, RAC1^S17N^ and CDC42^S17N^ mutants on actin stress fiber turnover and found no effect on stress fiber distribution and morphology in COS7 cells (Fig 7 and S10 Fig). Co-expression of each of the S17N mutants with wild-type RIT prevented stress fiber dissolution (S10 Fig), and expression of the kinase-dead PAK1 mutant K299A [59] together with RIT1 wildtype also reverted the phenotype of COS7 cells expressing RIT1 alone (compare Fig 6A and Fig 7). These data suggest that RIT1, RAC1/CDC42, and PAK1 may act in a signaling module regulating stress fiber turnover and cytoskeletal re-organization.

### RIT1 and NS-associated RIT1 mutants increase cellular invasion and migration

**Fig 8:**
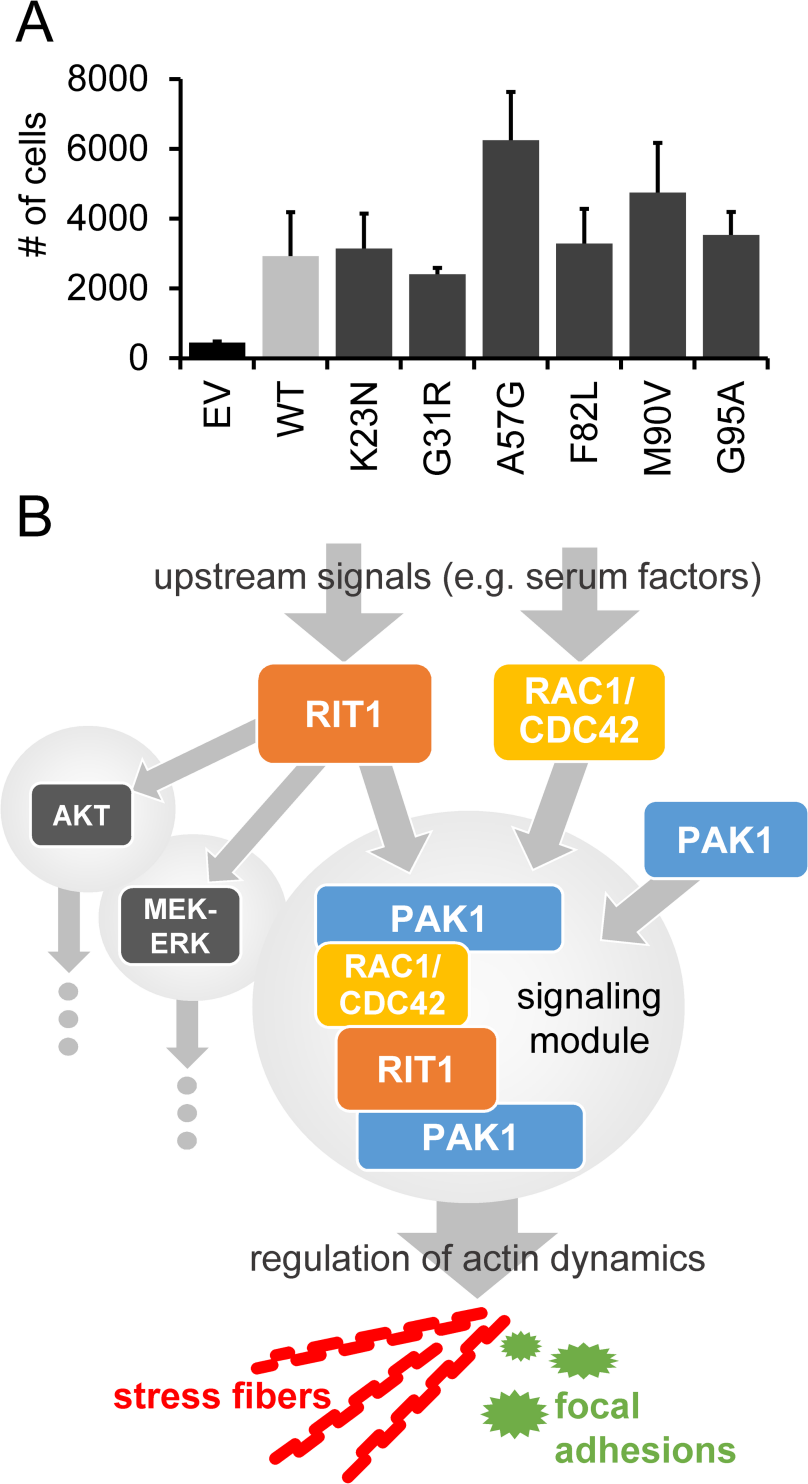
RIT1 enhances migration and invasive capabilities of cells by regulating actin dynamics. (A) Transwell assay of HEK293T cells transiently transfected with the indicated constructs and kept under serum starvation overnight. Cells were seeded in serum-free medium into an upper compartment of a transwell chamber. Cells passed a growth factor-reduced matrigel as a barrier towards a lower compartment with medium containing 10% serum as a chemoattractant (see materials and methods for details). After 48 hours, only cells located in the lower compartment were detached and counted by flow cytometry; a constant number of counting beads ensured comparability. The graphs show the mean ± SD of cell counts from three independent experiments. One-way ANOVA between groups: *P* < 0.001. *Post hoc P* values calculated by pairwise *t*-tests and Bonferroni correction were not significant. (B) Model depicting RIT1’s function in the regulation of cytoskeletal dynamics. Extracellular stimuli induce the formation of various parallel signaling hubs, such as PI3K-AKT, MEK-ERK and RAC1/CDC42-PAK1 modules. RIT1 can stimulate the activation of AKT and MEK-ERK signaling cascades. On the other hand, RIT1 interacts with PAK1 and RAC1/CDC42 to regulate actin-dependent structures, such as stress fibers and focal adhesions that may positively influence cell migration and adhesion.

PAK1 was found to control protrusive activity and cell migration [60] and increase adhesion turnover [56]. We studied the consequences of RIT1 on invasion and migration of HEK293T by using transwell migration assay. Transfection efficiency was about 99% for each *RIT1* construct (S11 Fig). Cells transiently expressing RIT1 wildtype showed increased migration and/or invasive and chemotactic behavior compared to cells transfected with empty vector (Fig 8A); the same applies to cells expressing any of the RIT1 mutants (ANOVA *P* <0.001; Fig 8A). Taken together, our findings suggest that RIT1 may regulate actin-depending cell motility.

## Discussion

The recent identification of germline *RIT1* mutations in patients with NS and somatic *RIT1* mutations in myeloid malignancies and lung adenomacarcinoma [37, 61] raises the question on the functional consequences of these genetic alterations. Here, we confirm activating effects of disease-associated *RIT1* mutations on MEK-ERK signaling. More importantly, we identified a direct protein-protein interaction between PAK1 and RIT1, found the RHO GTPases RAC1 and/or CDC42 to be in complex with RIT1 and showed that NS-associated amino acid substitutions enhance all of these interactions. This signaling complex turned out to facilitate the turnover of actin stress fibers, demonstrating a role of RIT1 in actin dynamics (Fig 8B).

### Consequences of NS-associated RIT1 mutants on MEK-ERK and PI3K-AKT signaling pathways

Rit1 shares effector molecules with Ras, such as Raf1 and the p110 catalytic subunit of PI3K [36]. We detected binding of RIT1 to PIK3CA and also to RALGDS and PLCE1. We show that RIT1 promotes phosphorylation of ERK1/2 in HEK293T cells upon serum factor stimulation, which is further intensified by the six NS-associated RIT1 mutants tested here. Similarly, EGF-induced ERK activation was previously found to be enhanced and/or sustained in T-REx293T cells expressing NS-associated RIT1 mutants [21], and ectopic expression of NS- and cancer-related RIT1 mutants in PC6 cells induced phosphorylation of both MEK and ERK [25, 37]. Collectively, the data indicate that RIT1 is a positive modulator of RAF-MEK-ERK signaling, and NS-associated mutants cause sustained MEK-ERK activation upon stimulation rather than a constitutive hyper-activation.

RIT1 binding to PIK3CA was enhanced upon serum stimulation of HEK293T cells suggesting that the RIT1-PI3K interaction is physiologically relevant. However, we could not detect any effect of the RIT1 mutants on AKT phosphorylation levels downstream of PI3K that is in line with previous experiments, in which expression of Rit1 in PC6 cells and the human neuroblastoma cell line SH-SY5Y did not induce AKT activation [8, 62]. In another study, however, oncogenic RIT1 mutants, including p.A77S and p.M90I also found in NS, stimulated phosphorylation of AKT in PC6 cells [37]. RIT1-dependent AKT activation in adult hippocampal neuronal precursor cells suggested a role of this signaling cascade in neurogenesis [63]. Taken together, data on AKT activation by RIT1 are controversial, and functional relevance of RIT1-PIK3-AKT signaling may significantly vary between different cell types.

### RIT1 is a regulator of actin dynamics

We identified direct binding of RIT1 to PAK1 as well as RAC1 and CDC42. Formation of these protein complexes is stimulated by serum factors, and all disease-associated RIT1 amino acid substitutions enhance complex formation and uncouple binding of RIT1 to PAK1 and RAC1/CDC42 from extracellular stimuli. Our results strengthen previous data demonstrating Rit1 in a complex with Rac1 or Cdc42 [44, 45], both of which are master regulators of the actin cytoskeleton [49, 50]. PAK1 acts directly downstream of the two GTPases and primarily controls polymerization of actin structures [48]. Expression of activated forms of Pak1 resulted in loss of stress fibers [37, 51, 58, 64], disassembly of focal adhesions [51, 52], and adhesion turnover by paxillin phosphorylation [56]. Here we show that expression of wild-type RIT1 and NS-associated RIT1 mutants leads to dissolution of stress fibers and a shift from mature to nascent paxillin-containing focal adhesions in COS7 cells. Moreover, co-expression of RIT1 with a dominant-negative form of CDC42, RAC1 or PAK1 blocked the phenotypic effects on stress fibers induced by RIT1. This data suggests that RIT1, PAK1, RAC1/CDC42 act together in a signaling module controlling actin-dependent processes (Fig 8B). Indeed, Rit1 p.Q79L has been previously demonstrated to initiate cytoskeletal changes, such as membrane extensions with ruffle-like structures in NIH 3T3 cells and the formation of neurite-like extensions in PC6 and SH-SY5Y cells [42, 62].

Reorganization of stress fibers and focal adhesion turnover are fundamental processes for cell adhesion and motility [65]. Both Rac and Cdc42 and their effector Pak play a key role in regulating cell migration [43]. For example, Pak1 operates downstream of Rac to regulate F-actin turnover in the lamellipodium and spatially and temporally organizes the interplay between actin, myosin and focal adhesion dynamics [60, 66]. Thus, the observed trend toward enhanced migration of COS7 cells expressing either wild-type RIT1 or a NS-associated RIT1 mutant and the finding of RIT1 as positive regulator of neurite outgrowth [6, 62] suggest an important role of this small GTPase in coordinating the cytoskeletal system during cell migration (Fig 8B). However, the impact of NS-associated RIT1 mutants on stress fiber dissolution, maturation of focal adhesions and cell migration/invasion was similar to wild-type RIT1 suggesting that (I) most mutations do not exert a gain-of-function effect in this specific cellular context or (II) ectopic overexpression of wild-type RIT1 is sufficient to induce the observed cellular effects.

### Dysregulated F-actin dynamics as novel pathomechanism in RASopathies

NS belongs to the RASopathies, a group of syndromes caused by germline gain-of-function mutations in genes encoding components or regulators of the RAS-MAPK pathway. Thus, dysregulated RAS-MEK-ERK signaling is the well-established biological mechanism shared among these disorders [33, 67]. Our data point to a novel aspect in the molecular pathogenesis of RASopathies as we uncovered RIT1 as regulator of cytoskeletal changes through the RHO GTPases RAC1 and CDC42 and their effector PAK1. This is in line with recent findings reported by Martinelli et al. (2018). They demonstrated that specific *CDC42* mutations are associated with a RASopathy-like phenotype. Cells expressing the CDC42 mutants p.Cys81Phe, p.Ser83Pro and p.Ala159Val showed enhanced polarized migration and cell growth [68]. Similarly, *RAC1* germline missense mutations cause varying developmental disorders depending on whether they act as dominant-negative or gain-of-function alleles. *RAC1* mutations had variable effects on fibroblast morphology during spreading; for example, expression of RAC1^Tyr64Asp^ resulted in a greater proportion of cells with lamellipodia and ruffles, similar to constitutive active RAC1 [69]. Other proteins encoded by disease genes for NS [70] have been also linked to the RHO GTPase pathway and F-actin dynamics. For example, RRAS promotes actin polymerization and focal contact formation via the regulation of Rac activity [39–41]. RRAS also associates with Pak1 and the actin binding protein filamin A, thereby mediating cytoskeletal reorganization [71], cell migration and integrin activation [72]. The two RASopathy-related proteins SHOC2 and PPP1CB regulate cell migration [73, 74], and SOS1, a dual guanine nucleotide exchange factor for Ras and Rac, induces Rac-dependent actin remodeling [75]. By introducing the NS-associated *PTPN11* mutation p.N308D in *Xenopus*, the encoded protein phosphatase SHP-2 was found to regulate actin dynamics in the developing heart *in vivo* [76]. Similarly, valve cell migration was increased by expression of the SHP-2 mutant Q510E [77]. These and our data indicate that in addition to deregulated RAS-MEK-ERK signaling, dysfunction in actin dynamics and associated processes are pathophysiological mechanisms underlying the group of RASopathies. Thus, altered actin-dependent processes such as cell spreading, adhesion and migration may explain certain clinical features in patients with NS [38, 68].

## Materials and methods

### GTPase pull down assay

The RAS-binding domain (RBD) of PI3K (PIK3CA) (amino acids 127–314), the RAS-association (RA) domain of RALGDS (amino acids 777–872), the RA domain of PLC1 (PLCE1) (amino acids 2130–2240) and the CRIB domain of PAK1 (amino acids 58–141) were used to specifically pull down GTP-bound RIT1 from cell extracts. Preparation of GST-RBD/RA/CRIB beads, cell lysis and precipitation of GTP-bound GTPases have been described previously [78].

### Co-immunoprecipitation

Transiently transfected HEK293T cells were cultured as indicated in the respective experiment. Cells were lysed in ice-cold co-immunoprecipitation buffer [150 mM NaCl, 50 mM HEPES pH 7.5, 0.2% Nonidet P40, 1 mM Glycerol; supplemented with complete Mini Protease Inhibitors (Roche)], and cell lysates were clarified by centrifugation. RAC1 immunoprecipitation shown in S5B Fig was performed with an alternative co-immunoprecipitation buffer [50 mM Tris-HCl pH 8, 120 mM NaCl, 1 mM EDTA, 0.5% Nonidet P40; supplemented with complete Mini Protease Inhibitors (Roche)]. After removing small aliquots (total cell lysates), supernatants were pre-cleared for 45 min with 20 μl Protein A agarose beads (Roche) or Protein G sepharose beads (GE Healthcare) on a rotator at 4°C followed by centrifugation. 1–2 μg of antigen-specific primary antibody or an IgG isotype control antibody were added to the supernatants. In some experiments, the pre-cleared lysates were split into two aliquots and two different antigen-specific antibodies were used for immunoprecipitation. Solutions were incubated on an end-over-end rotator overnight at 4°C. Subsequently, 20 μl Protein A agarose beads (Roche) for antibodies raised in rabbit or 20 μl Protein G sepharose beads (GE Healthcare) for mouse IgG antibodies were added and mixtures were incubated for 1 h at 4°C on a rotator. For ectopically expressed proteins, the supernatants were transferred to either 20 μl EZview Red Anti-Myc Affinity Gel, 20 μl EZview Red Anti-FLAG Affinity Gel (Sigma-Aldrich) or GFP-Trap A (Chromotek) and incubated for 2 h at 4°C on a rotator. Precipitates were collected by repeated centrifugation and washing with co-immunoprecipitation buffer (50 mM Tris-HCl, pH 7.4; 150 mM NaCl), resuspended in sample buffer (33% glycerol, 80 mM Tris-HCl, pH 6.8, 0.3 M Dithiothreitol, 6.7% sodium dodecyl sulphate, 0.1% bromophenol blue) and subjected to SDS-PAGE and immunoblotting. PAK1 and CDC42 co-immunoprecipitations shown in S3B, S5A and S6D Figs were performed with magnetic Dynabeads Protein G (Thermo Fisher Scientific). Therefore, 1–2 μg of anti-PAK1 or anti-CDC42 antibodies were bound to Dynabeads on a rotator for 10 min at room temperature followed by a washing step with PBST. Cells were lysed in 500 μl mRIPA buffer [50 mM HEPES pH 7.5, 150 mM NaCl, 1% Nonidet P40, 0.5% sodium deoxycholate, 0.05% sodium dodecyl sulphate, 1 mM EDTA; supplemented with complete Mini Protease Inhibitors and PhosphoStop (Roche)] for 15 min at 4°C, and cell debris was cleared by centrifugation for 20 min. After removing an aliquot (total cell lysate), the remaining supernatant was precleared with Dynabeads Protein G for 1 h at 4°C followed by incubation with the antibody-bound Dynabeads overnight at 4°C on a rotator. Next day, the Dynabeads were pelleted and washed five times with mRIPA buffer. The bound target proteins were eluted by resuspending the beads in 25 μl 1x sample buffer and subjected to SDS-PAGE and immunoblotting.

### *In-vitro* binding assay

GST-PAK[CRIB], GST-CDC42 (amino acids 1–178) and GST-RAC1 (amino acids 1–181) fusion proteins were isolated from *E*. *coli* strain BL21 transformed with pGEX-2TK-PAK[CRIB], pGEX-4T3-CDC42 and pGEX-4T3-RAC1, respectively, as described previously [78]. GST fusion proteins were coupled to glutathione-bound agarose beads (CAS 64-17-5; Macherey-Nagel), washed in wash buffer (50 mM Tris-HCl, pH 8.0; 50 mM NaCl; 5 mM MgCl_2_) and finally diluted to 50% slurry with wash buffer. His-RIT1^WT^, His-RIT1^A57G^ and His-RIT1^F82L^ (amino acids 1–201 each) were purified from *E*. *coli* BL21 cells transformed with pET151/D-TOPO-RIT1^WT^, pET151/D-TOPO-RIT1^A57G^ and pET151/D-TOPO-RIT1^F82L^, respectively, by using the Champion pET Directional TOPO Expression Kit (K151-01, Life Technologies) according to manufacturer’s instructions. Depending on the experimental design, recombinant His-RIT1^WT/A57G/F82L^, GST-CDC42 and GST-RAC1 were coupled to GDP (G7127, Sigma-Aldrich) or non-hydrolyzable GTPγS (G8634, Sigma-Aldrich) for 30 min by incubation with 20 mM Tris-HCl, 100 mM NaCl, 0.1% Triton X-100, 1 mM DTT, 2.5 mM EDTA and 1 mM of GDP or GTPγS at room temperature. Reaction was stopped by adding 20 mM MgCl_2_. GST-PAK[CRIB], GDP/GTPγS-loaded GST-CDC42 and GDP/GTPγS-loaded GST-RAC1 fusion proteins coupled to glutathione-bound agarose beads were mixed with different concentrations of GDP/GTPγS-loaded His-RIT1^WT/A57G/F82L^ protein—as indicated in the respective experiment—in binding buffer (20 mM Tris-HCl, 100 mM NaCl, 0.1% Triton X-100, 1 mM DTT, 100 mM MgCl_2_). After incubation for 30 min at room temperature, precipitates were collected by centrifugation and washed with binding buffer (4 times) and proteins were analyzed by western blotting. Purified GST-SAPAP2 (NM_004745.4) (expressed from plasmid pGEX-6P-1) was a gift from Stefan Kindler (Institute of Human Genetics, University Medical Center Hamburg-Eppendorf, Hamburg, Germany).

### Immunocytochemistry

Coverslips were coated with 10 μg/ml collagen type I (#08–115, EMD Millipore) in PBS for 1 hour at room temperature. Excess collagen was removed, COS7 cells were seeded on coverslips, transfected with expression constructs and serum-deprived overnight. Subsequently, cells were rinsed with PBS, fixed with 4% paraformaldehyde (Sigma-Aldrich) in PBS and washed three times with PBS. After treatment with permeabilization/blocking solution (2% BSA, 3% goat serum, 0.5% Nonidet P40 in PBS), cells were incubated in antibody solution (3% goat serum and 0.1% Nonidet P40 in PBS) containing appropriate primary antibodies. Cells were washed with PBS and incubated with Fluorophore-conjugated secondary antibodies or Texas Red-X phalloidin (Life Technologies) in antibody solution. After extensive washing with PBS cells were embedded in ProLong Diamond Antifade Mountant with DAPI (P36962, Life Technologies) on microscopic slides. Cells were examined in epifluorescence mode of an Olympus cell tool TIRFM system equipped with a 60x oil immersion objective lens and pictures were taken of representative cells to visualize the observed morphological changes. Phalloidin-stained cells were categorized in two different groups: cells with normal and cells with reduced or absent actin stress fibers (exemplary photographs are shown in S8B Fig). A minimum of 50 cells per dataset were analyzed. The number of paxillin-positive (dot-like and stripe-like) structures per cell was determined in a minimum of 30 cells per dataset by using ImageJ software (NIH; http://rsb.info.nih.gov/ij/index.html).

### Transwell migration assay

Cell invasion was investigated by using Costar Transwell cell culture inserts for 24 well plates with 8 μm pores (Corning), which were coated with 100 μg/cm^2^ growth factor reduced Matrigel Basement Membrane Matrix (Corning) according to manufacturer´s instructions. Briefly, 10^5^ HEK293T cells transiently expressing various RIT1 protein variants were seeded in transwell upper compartment in a volume of 100 μl of serum-free DMEM. For chemo attraction, 600 μl of DMEM supplemented with 10% FBS were added into the lower compartment. 48 h later cells that moved to the lower compartment were carefully detached by trypsinization and transferred to FACS tubes. Cell numbers were determined by flow cytometry using AccuCheck Counting Beads (Thermo Fisher Scientific).

### Flow cytometry

Transfection efficiency was determined by staining of cells with anti-HA-fluorescein antibody and quantification of HA-fluorescein positive cells. To determine cell numbers, detached cells were washed twice with PBS. To each FACS tube, 20 ml of AccuCheck Counting Beads (Thermo Fisher Scientific) was added. Flow cytometry was carried out using a FACS Calibur (BD Biosciences). In each sample, 3000 beads were acquired and the number of cells, which were collected in the meanwhile, was determined using CellQuestPro Software (BD Biosciences).

### Data analyses and statistics

Signals on autoradiographs were quantified by densitometric analysis using the ImageJ software (NIH; http://rsb.info.nih.gov/ij/index.html). Quantitative data are presented as the mean ± standard deviation (SD) performed by GraphPad prism7 software (Instat, GraphPad Software). Statistical significance was assessed by Student's *t*-test for pairwise comparisons or one-way ANOVA for multiple comparisons. In the latter case, statistically significant differences were identified by *post hoc* analysis using Student's *t*-test followed by Bonferroni correction. Data shown in graphs of Fig 1B and S1A Fig were derived from various independent immunoblottings/autoradiographs; thus, ANOVA was not applicable for all RIT1 protein variants without normalization to RIT1 wildtype. Therefore, data were split into data sets consisting of RIT1 wildtype and one RIT mutant in each case (both of which were derived from the same experiment/autoradiograph), and separate graphs for these data sets are presented (Fig 1B and S1A Fig). For the same reason, data shown in Fig 1C were split into two data sets consisting of RIT1 wildtype and three RIT1 mutants in each case (all were derived from the same immunoblotting/autoradiograph). Data shown in Fig 3C and S3A Fig were derived from two independent experiments; therefore, ANOVA was not applicable as it needs at least three data points. Assessments were considered significant at *P* value <0.05.

## Supporting information

S1 Text Additional materials and methods are available in Supporting Information. (DOCX)

S1 Fig Expression of the RIT1 mutants enhance phosphorylation of ERK1/2 upon serum stimulation. (A and B) HEK293T cells were transfected with empty vector (EV) and constructs expressing HA-RIT1 wildtype (WT), HA-RIT1 p.K23N, p.G31R, p.F82L, p.M90V or p.G95A as indicated. Cells were cultured under serum-starved condition (0.1% serum; 0 min) and serum-starved condition followed by 5, 15, or 30 min stimulation with 20% serum. Total cell lysates were analyzed by immunoblotting using anti-phospho-ERK1/2 (pERK1/2) (A and B) and anti-ERK1/2 (ERK1/2) antibodies (A). Expression of RIT1 protein variants was monitored by immunoblotting using anti-HA antibody, and anti-GAPDH antibody was applied to control for equal loading (A). Data shown are representative of three independent experiments. (B) Immunoblots from three independent experiments (Exp.) demonstrate that the RIT1 p.G95A mutant stimulates ERK1/2 phosphorylation under serum-starved condition (0 min). The immunoblot shown in Exp. 1 is the same as the one in Fig 1B (most upper blot on the right). Autoradiographic signals were quantified by scanning densitometry. Levels of phosphorylated ERK1/2 were normalized relative to amounts of total ERK1/2. To conserve the relative variance of the samples, values for RIT wildtype and mutants were divided by the mean of the wildtype samples [79]. Graphs show relative phosphorylation levels (arbitrary units) upon serum starvation (0 min) and after 5, 15, and 30 min serum stimulation in cells expressing RIT1 wildtype (WT), RIT1 p.K23N, p.G31R or p.M90V. The mean of three independent experiments ± SD is given. Unpaired *t*-tests were used to determine statistical significance (*, *P* <0.05; ***, *P* <0.001). (C) HEK293T cells were transfected with empty vector (EV) or HA-tagged RIT1 expression constructs (wildtype [WT] and p.G31R) as indicated and cultured under steady-state condition (10% serum). Total cell lysates were analyzed as described in (A). Two independent experiments (Exp. #1 and #2) are shown.
(TIF)

S2 Fig AKT phosphorylation at serine 473 and threonine 308 upon expression of RIT1 wildtype and mutants. (A) HEK293T cells were transfected with empty vector (EV) and constructs expressing HA-RIT1 wildtype (WT), HA-RIT1 p.A57G, p.F82L or p.G95A as indicated. Cells were cultured under serum-starved condition (0.1% serum; 0 min) and serum-starved condition followed by 5, 15, or 30 min stimulation with 20% serum. Total cell lysates were analyzed by immunoblotting using anti-phospho-AKT^Ser473^ (pAKT^Ser473^) and anti-AKT (AKT) antibodies. Expression of RIT1 protein variants was monitored by immunoblotting using anti-HA antibody, and anti-GAPDH antibody was used to control for equal loading. Data shown are representative of three independent experiments. (B) HEK293T cells were transfected with empty vector (EV) or a construct expressing HA-RIT1 wildtype (WT), cultured under serum-starved condition (0.1% serum; 0 min) and serum-starved condition followed by 5, 15, or 30 min stimulation with 10 ng/ml EGF. Total cell lysates were analyzed by immunoblotting using anti-phospho-AKT^Thr308^ (pAKT^Thr308^) and anti-AKT (AKT) antibodies. Expression of HA-tagged RIT1 protein was monitored by immunoblotting using anti-HA antibody, and anti-GAPDH antibody was used to control for equal loading. Data shown are representative of three independent experiments.
(TIF)

S3 Fig RIT1 amino acid changes stimulate binding of RIT1 to PAK1. (A and B) HEK293T cells were transfected with empty vector (EV) and RIT1 expression constructs as indicated and cultured under serum deprivation (0.1% serum, A) or basal condition (10% serum, B). Endogenous PAK1 was precipitated with an anti-PAK1 antibody [IP: PAK1 (#2) in (A) from the same extract as shown in Fig 3C; IP: PAK1 (#1) in (B)], and co-precipitated HA-RIT1 was detected using an anti-HA antibody. Enrichment of PAK1 in the precipitates was demonstrated with an anti-PAK1 antibody. The star indicates the heavy chain of the antibody used for precipitation. The amount of HA-RIT1 and PAK1 in total cell lysates (TCL) was monitored by immunoblotting using an anti-HA antibody and an anti-PAK1 antibody, respectively. Data shown are representative of two (A) or three (B) independent experiments. Autoradiographic signals were quantified by scanning densitometry. Levels of co-IPed HA-RIT1 was double-normalized relative to amounts of immunoprecipitated PAK1 and HA-RIT1 in total cell lysates. To conserve the relative variance of the samples, values for RIT1 wildtype and RIT1 mutants were divided by the mean of the wildtype samples [79]. The graphs show the relative amount (arbitrary units) of co-precipitated RIT1 protein variants. The mean of two (A) or three (B) independent experiments ± SD is given, respectively. (A) Unpaired *t*-tests were used to determine statistical significance. ns, not significant. (B) One-way ANOVA between groups: *P* < 0.05; ns, not significant.
(TIF)

S4 Fig HA-RIT1 p.G95A stimulates binding of RIT1 to PAK1, but PAK4 is not an interaction partner of RIT1. (A) Detection of endogenous PAK1 in serum-starved HEK293T, HeLa and COS7 cells after cell lysis and immunoblotting by using an anti-PAK1 antibody (#1). PAK1 expression is high in HEK293T and weak in COS7 cells. (B) COS7 cells were transfected with HA-RIT1 p.G95A expression construct and cultured under serum deprivation (0.1% serum). Endogenous PAK1 was precipitated with an anti-PAK1 antibody [IP: PAK1 (#3)]. As IP control, an irrelevant isotype-matched antibody (anti-pSMAD2 antibody) was used (IP: ctrl). Enrichment of PAK1 in the precipitates and the amount of endogenous PAK1 in TCL was demonstrated with an anti-PAK1 antibody (#2). Co-precipitated HA-RIT1 and expression of HA-RIT1 in total cell lysates (TCL) was detected by an anti-HA antibody. (C) COS7 cells were transfected with empty vector (EV), RIT1 wildtype or p.G95A expression constructs as indicated and cultured under serum deprivation (0.1% serum). Endogenous PAK1 was precipitated with an anti-PAK1 antibody [IP: PAK1 (#1)]. Enrichment of PAK1 in the precipitates and the amount of endogenous PAK1 in TCL was demonstrated with an anti-PAK1 antibody (#1). Co-precipitated HA-RIT1 and expression of HA-RIT1 in TCL was detected by immunoblotting using an anti-HA antibody. Juxtaposed autoradiographs derive from the same western blotting membrane. (D) HEK293T cells expressing GFP-PAK4 and either HA-tagged RIT1 WT or the HA-RIT1 p.G95A mutant were cultured under serum deprivation (0.1% serum). GFP-PAK4 was precipitated by GFP trap. Enrichment of GFP-PAK4 (GFP trap) is shown by direct comparison of precipitates and TCL. HA-RIT1 was detected by using an anti-HA antibody. As positive IP control, co-precipitated endogenous CDC42 was detected by an anti-CDC42 antibody. Data are representative for three independent experiments.
(TIF)

S5 Fig Interaction of CDC42 and RAC1 with RIT1 wildtype and mutants in HEK293T cells cultured under basal condition. (A and B) HEK293T cells were transfected with empty vector (EV) and RIT1 expression constructs as indicated and cultured under basal condition (10% serum). Endogenous CDC42 and RAC1 were immunoprecipitated with an anti-CDC42 antibody [CDC42 (#3)] (A) and an anti-RAC1 antibody (B), respectively. Co-precipitated HA-RIT1 and HA-RIT in total cell lysates (TCL) was detected by an anti-HA antibody. Enrichment of CDC42 and RAC1 in the precipitates and amount of endogenous CDC42 and RAC1 in TCL was demonstrated with an anti-CDC42 antibody [CDC42 (#4)] (A) and an anti-RAC1/2/3 antibody (B), respectively. Data shown for CDC42 and RAC1 are representative of three (A) and four (B) independent experiments, respectively. Autoradiographic signals were quantified by scanning densitometry. The amount of co-precipitated HA-RIT1 was double-normalized relative to amounts of immunoprecipitated CDC42/RAC1 and HA-RIT1 in total cell lysates. To conserve the relative variance of the samples, values for RIT1 wildtype and RIT1 mutants were divided by the mean of the wildtype samples [79]. The graphs show the relative amount (in arbitrary units) of co-precipitated RIT1 protein variants. The mean of three (A) and four (B) independent experiments ± SD is given. One-way ANOVA between groups: *P* < 0.05; ns, not significant.
(TIF)

S6 Fig RIT1 associates with CDC42 and RAC1, but not with RHOA. (A and B) HEK293T cells were co-transfected with MYC-empty vector (MYC-EV), MYC-CDC42 (A) or MYC-RAC1 (B) expression construct together with HA-empty vector (HA-EV), RIT1 wildtype (WT) or RIT1 p.G95A expression construct and cultured under serum deprivation (0.1% serum). Lysates were split for precipitation using either anti-MYC coupled beads (Trap MYC in A and B) or primary antibodies against CDC42 (IP CDC42 in A) and RAC1 (IP RAC1 in B). As IP control for primary antibodies an irrelevant isotype-matched antibody against V5 (V5) was used. Both in precipitates (IP, Trap) and total cell lysates (TCL), RIT1 protein was monitored by immunoblotting using an anti-HA antibody, RAC1 and CDC42 were detected by an anti-MYC antibody. Anti-GAPDH antibody was used to control for equal loading. Results are representative for two independent experiments. (C) HEK293T cells were co-transfected with EGFP-empty vector (EGFP-EV) or an EGFP-RHOA expression construct (EGFP-RHOA) together with HA-empty vector (HA-EV), RIT1 wildtype (WT) or RIT1 p.G95A expression construct and cultured under serum deprivation (0.1% serum). Enrichment of EGFP or EGFP-RHOA by EGFP trap (Trap EGFP) is shown by direct comparison of precipitates and total cell lysates (TCL). HA-RIT1 was detected by using an anti-HA antibody and EGFP and EGFP-RHOA by an anti-EGFP antibody. Asterisk indicates the light chain of the antibody used for precipitation. Data are representative for three independent experiments. (D) Endogenous CDC42 of stably transfected Flp-In 293 cells expressing RIT1^G31R^ or RIT1^A57G^ was immoprecipitated with an anti-CDC42 antibody [IP: CDC42 (#5)]. Endogenous CDC42 protein in total cell lysates (TCL) and precipitates was detected with an anti-CDC42 antibody [WB: CDC42 (#4)]. Co-precipitated HA-RIT1 and HA-RIT1 in TCL was detected by an anti-HA antibody. As a control, an isotype-matched non-specific anti-IgG rabbit antibody (IgG) was used for immunoprecipitation. The asterisk indicates the light chain of the antibody used for precipitation.
(TIF)

S7 Fig RIT1 interacts with CDC42 and RAC1 *in vitro*. Recombinant His-tagged RIT1 wildtype (0.5 μM) was loaded with GDP or non-hydrolyzable GTPγS as indicated and incubated with 1 μM GST-CDC42 (loaded with GDP or GTPγS) (A) or GST-RAC1 (loaded with GDP or GTPγS) (B). Recombinant GST (1 μM) and GST-PAK[CRIB] (1 μM) were used as negative and positive control, respectively. Glutathione agarose-coupled GST fusion proteins were precipitated and samples were analyzed by immunoblotting using an anti-His antibody (precipitates and input) and an anti-GST antibody (precipitates). Data shown are representative of three independent experiments.
(TIF)

S8 Fig F-actin and paxillin staining of untransfected COS7 and HEK293T cells and categorization of RIT1 wildtype expressing COS7 cells by the amount of actin stress fibers. (A) COS7 and HEK293T cells were plated on collagen-coated glass slides and kept under serum starvation overnight. F-actin was visualized using Texas Red-X Phalloidin. Paxillin was stained using mouse anti-paxillin antibody and Alexa Fluor488-conjugated anti-mouse antibody. Nuclear DNA was labeled by DAPI. White boxes indicate magnified parts of specimen shown on the very right-hand side. Scale bar, 10 μm. (B) COS7 cells were plated onto collagen-coated coverslips, transiently transfected with the HA-RIT^WT^ construct and serum-starved overnight. Cells were stained with mouse anti-HA-antibody followed by anti-mouse Alexa Fluor 488-conjugated antibody, Texas Red-X Phalloidin for actin distribution and DAPI for nucleus staining. White boxes indicate magnified parts of specimen shown on the right-hand side. Scale bars, 10 μm. Exemplary images depicting cells with normal, reduced or absent actin stress fibers are shown.
(TIF)

S9 Fig Expression of RIT1 and NS-associated RIT1 mutants causes a shift from mature (stripe-like) to nascent (dot-like) paxillin-containing focal adhesions. (A) COS7 cells were plated on collagen-coated glass slides, transiently transfected with the indicated construct and kept under serum starvation overnight. HA-tagged RIT1 was stained by rabbit anti-HA antibody followed by anti-rabbit Alexa Fluor546-conjugated antibody. Paxillin was visualized using mouse anti-paxillin antibody and Alexa Fluor488-conjugated anti-mouse antibody. Nuclear DNA was labeled by DAPI. Scale bar, 10 μm. (B) The number of nascent (dot-like) and mature (stripe-like) paxillin-positive structures per cell was determined in at least 30 cells of each dataset.
(TIF)

S10 Fig Co-expression of dominant-negative CDC42^S17N^ or RAC1^S17N^ with RIT1 prevents loss of stress fibers. COS7 cells were plated on collagen-coated glass slides, transiently transfected with the indicated constructs and serum-starved overnight. MYC-tagged CDC42^S17N^ (A) and RAC1^S17N^ (B) were stained by mouse anti-MYC antibody followed by anti-mouse Alexa Fluor488-conjugated antibody. HA-tagged RIT1 was visualized using rabbit anti-HA antibody and anti-rabbit Alexa Fluor647-conjugated antibody. F-actin was stained by using Texas Red-X Phalloidin, and nuclear DNA was labeled by DAPI. The label “HA” indicates cell(s) not transfected with the RIT1 wildtype construct. For co-expression of RAC1^S17N^ and RIT1 wildtype two separate microscopic data series are shown (last two rows in B). White boxes indicate magnified parts of specimen shown on the very right-hand side. Scale bar, 10 μm.
(TIF)

S11 Fig Transfection efficiency of *RIT1* constructs in HEK293T cells used for transwell activation assay. HEK293T cells were transiently transfected with cDNA constructs expressing the indicated HA-tagged RIT1 protein variants and serum-starved overnight. Cells were stained with anti-HA-fluorescein antibody and counted by flow cytometry. Percentage of cells positive for HA-fluorescein staining is indicated for each construct.
(TIF)
