# Supplementary material for: RIT1 controls actin dynamics via complex formation with RAC1/CDC42 and PAK1
Source: PLoS Genet. 2018 May 7;14(5):e1007370. doi: 10.1371/journal.pgen.1007370 (PMC5937737; doi:10.1371/journal.pgen.1007370)
Supplement: S1 Text — (DOCX) [file pgen.1007370.s001.docx]

**Supporting Information: S1 Text**

**Materials and Methods**

**Plasmids**

Human RIT1 (NM_006912) in pCR4-TOPO was a gift from Yoko Aoki (Department of Medical Genetics, Tohoku University School of Medicine). The C-terminal FLAG tag was removed and RIT1 was subcloned into pMT2SM-HA, in frame with an N-terminal HA tag. NS-associated mutations (c.69A>C, c.91G>C, c.170C>G, c.244T>G, c.268A>G, c.284G>C) were introduced into the RIT1 cDNA by using the Quik Change II Site Directed Mutagenesis Kit (#200523; Agilent) according to manufacturer´s instructions. Coding sequences for amino acids 1-201 of RIT1^WT^, RIT1^A57G^ and RIT1^F82L^ were subcloned into plasmid pET151/D-TOPO (K151-01; Life Technologies); this vector contains a polyhistidine region. Human CDC42 (NM_044472) and human RAC1 (NM_006908) in pRK5-MYC contain an N-terminal MYC tag and were gifts from Anja Schmidt (University College London). Human RHOA (NM_001313941) in pEGFP-C3 with a C-terminal EGFP fusion and human PAK4 (NM_005884) in pcDNA-DEST47 (C-terminal GFP epitope) were gifts from Stefan Linder (Institute for Medical Microbiology, Virology and Hygiene, University Medical Center Hamburg-Eppendorf). The sequences encoding CDC42 amino acids 1-178 and RAC1 amino acids 1-181 were subcloned into pGEX-4T3; this vector contains an N-terminal GST fusion peptide. The vector pGEX-2TK containing the sequence encoding the Cdc42/Rac interactive binding motif (CRIB) of human PAK1 (NM_002576; amino acids 58-141) was a gift from M. Reza Ahmadian (Institute of Biochemistry and Molecular Biology II, Heinrich Heine University, Düsseldorf).

**Cell culture and transfection**

HEK293T and COS7 cells were cultured in Dulbecco´s modified Eagle medium (DMEM, Life Technologies) supplemented with 10% (v/v) fetal bovine serum (FBS; Sigma-Aldrich) and penicillin-streptomycin (100 U/ml and 100 μg/ml, respectively; Life Technologies) and incubated at 37°C in a humidified atmosphere with 5% CO_2_. Cells were transiently transfected with TurboFect (Thermo Fisher Scientific) according to the protocol provided. 4 hours post transfection, medium was replaced as indicated in the respective experiment.

**RIT1 stably expressing cells**

The coding sequence of HA-tagged RIT1^G31R^ and RIT1^A57G^ mutants was subcloned into the pcDNA5/FRT vector harboring an FRT site for Flp recombinase-mediated integration and a hygromycin resistance gene for selection of stable transfected cells. Flp-In 293 cells (Thermo Fisher Scientific) containing an integrated FRT site were cotransfected with a 9:1 ratio of pOG44:pcDNA5/FRT/HA-RIT1 plasmid DNA with TurboFect. Cells were selected with 200 µg/ml hygromycin B (EMD Millipore) according to the manufacturers’ instructions. As a positive control for successful transfection and selection the pcDNA5/FRT/CAT vector was used. Expression of HA-RIT1 mutant proteins and V5-CAT was verified by immunoblotting. Co-immunoprecipitation experiments were performed with mixed populations of hygromycin-resistant Flp-In 293 cells stably expressing HA-RIT1 mutant p.G31R or p.A57G.

**Antibodies and reagents**

The following primary antibodies were used: rabbit anti-AKT (#9272, Cell Signaling Technology), rabbit anti-phospho-AKT (pAKT^Ser473^; Ser 473, #9271, Cell Signaling Technology), rabbit anti-phospho-AKT (pAKT^Thr308^; Thr 308, #9275, Cell Signaling Technology), mouse anti-CDC42 (#610929, BD Biosciences; #1 immunoprecipitation in Fig 5A and 5B), rabbit anti-CDC42 (#2462, Cell Signaling Technology; #2 western blot detection in Fig 5A and 5B), mouse anti-CDC42 (#ACD03, Cytoskeleton; #3 immunoprecipitation in S5A Fig), rabbit anti-CDC42 (#ab187643, Abcam; #4 western blot detection in S5A and S6D Fig), rabbit anti-CDC42 (#M00119, Boster Biological Technology; #5 immunoprecipitation in S6D Fig), mouse anti-EGFP (#902601, Bio Legend), mouse anti-c-myc-HRP (#11814150001, Roche), mouse anti-glycerinaldehyd-3-phosphat-dehydrogenase (GAPDH; ab8245, Abcam), anti-GST HRP conjugate (RPN1236, GE Healthcare), rat anti-HA-fluorescein, high affinity antibody (clone 3F10; #11988506001; Roche; FACS analysis), rat anti-HA-HRP (#12013819001, Roche), rabbit anti-p21-activated kinase [PAK1; #2602, Cell Signaling Technology (#1 in Fig 3B and 3C and S3B, S4A and S4C Fig), sc-881, Santa Cruz (#2 in S3A and S4B Fig) and #71-9300, Thermo Fisher Scientific (#3 in S4B Fig) co-immunoprecipitation and western blot detection as indicated], mouse anti-polyHistidine (H1029, Sigma-Aldrich), rabbit anti-p42/44 MAP kinase (ERK1/2; #9102, Cell Signaling Technology), rabbit anti-phospho-p42/44 MAP kinase (pERK1/2; Thr202/Tyr204; #9101, Cell Signaling Technology), mouse anti-RAC1 (#610650, BD Biosciences; co-immunoprecipitation), rabbit anti-RAC1/2/3 (#2465, Cell Signaling Technology; western blot detection), rabbit anti-phospho-SMAD2 (#3104, Cell Signaling Technology), mouse anti-V5 antibody (R960-25, Life Technologies). Secondary horseradish peroxidase (HRP)-coupled anti-rabbit (NA934V) and anti-mouse (NA931V) antibodies were from GE Healthcare. To detect precipitated PAK1 by western blotting, a light chain-specific HRP-coupled secondary antibody (#211-032-171, Dianova) was used. For immunocytochemistry: mouse anti-c-myc (Clone 9E10; M5546, Sigma-Aldrich), rabbit anti-HA (H6908, Sigma-Aldrich), mouse anti-HA (MMS-101P, Bio Legend), mouse anti-paxillin (#610052, BD Biosciences), Texas Red-X phalloidin (T7471, Life Technologies), Alexa Fluor488 goat anti-mouse (A11001, Life Technologies), Alexa Fluor488 goat anti-rabbit (A11008, Life Technologies), Alexa Fluor546 goat anti-rabbit (A11010, Life Technologies) and Alexa Fluor647 goat anti-rabbit (A32733, Life Technologies).
