# Supplementary figures and images for: RIT1 controls actin dynamics via complex formation with RAC1/CDC42 and PAK1
Source: PLoS Genet. 2018 May 7;14(5):e1007370. doi: 10.1371/journal.pgen.1007370 (PMC5937737; doi:10.1371/journal.pgen.1007370)

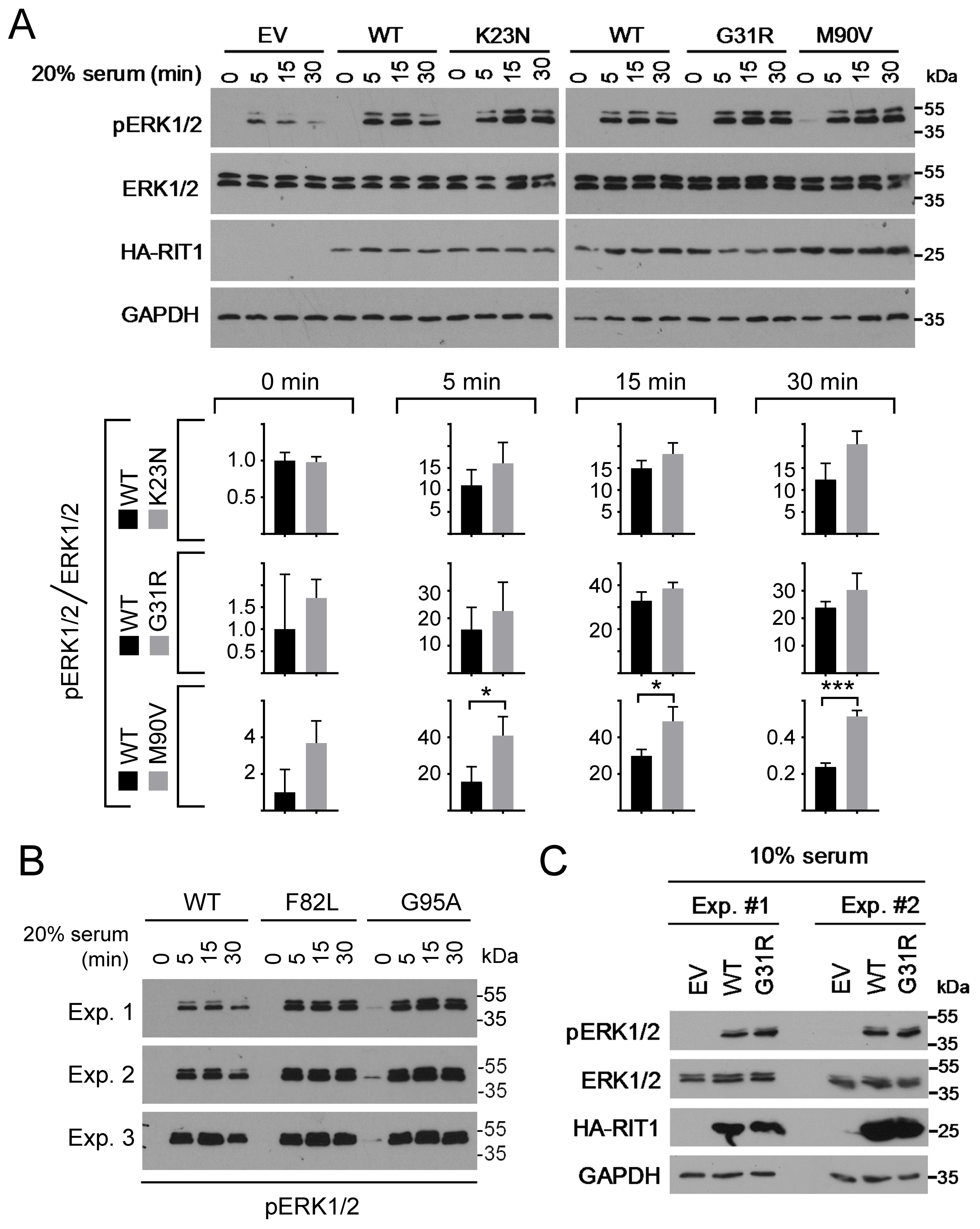

Supplement: S1 Fig — (A and B) HEK293T cells were transfected with empty vector (EV) and constructs expressing HA-RIT1 wildtype (WT), HA-RIT1 p.K23N, p.G31R, p.F82L, p.M90V or p.G95A as indicated. Cells were cultured under serum-starved condition (0.1% serum; 0 min) and serum-starved condition followed by 5, 15, or 30 min stimulation with 20% serum. Total cell lysates were analyzed by immunoblotting using anti-phospho-ERK1/2 (pERK1/2) (A and B) and anti-ERK1/2 (ERK1/2) antibodies (A). Expression of RIT1 protein variants was monitored by immunoblotting using anti-HA antibody, and anti-GAPDH antibody was applied to control for equal loading (A). Data shown are representative of three independent experiments. (B) Immunoblots from three independent experiments (Exp.) demonstrate that the RIT1 p.G95A mutant stimulates ERK1/2 phosphorylation under serum-starved condition (0 min). The immunoblot shown in Exp. 1 is the same as the one in Fig 1B (most upper blot on the right). Autoradiographic signals were quantified by scanning densitometry. Levels of phosphorylated ERK1/2 were normalized relative to amounts of total ERK1/2. To conserve the relative variance of the samples, values for RIT wildtype and mutants were divided by the mean of the wildtype samples [79]. Graphs show relative phosphorylation levels (arbitrary units) upon serum starvation (0 min) and after 5, 15, and 30 min serum stimulation in cells expressing RIT1 wildtype (WT), RIT1 p.K23N, p.G31R or p.M90V. The mean of three independent experiments ± SD is given. Unpaired t-tests were used to determine statistical significance (*, P <0.05; ***, P <0.001). (C) HEK293T cells were transfected with empty vector (EV) or HA-tagged RIT1 expression constructs (wildtype [WT] and p.G31R) as indicated and cultured under steady-state condition (10% serum). Total cell lysates were analyzed as described in (A). Two independent experiments (Exp. #1 and #2) are shown. (TIF) [file pgen.1007370.s002.tif]

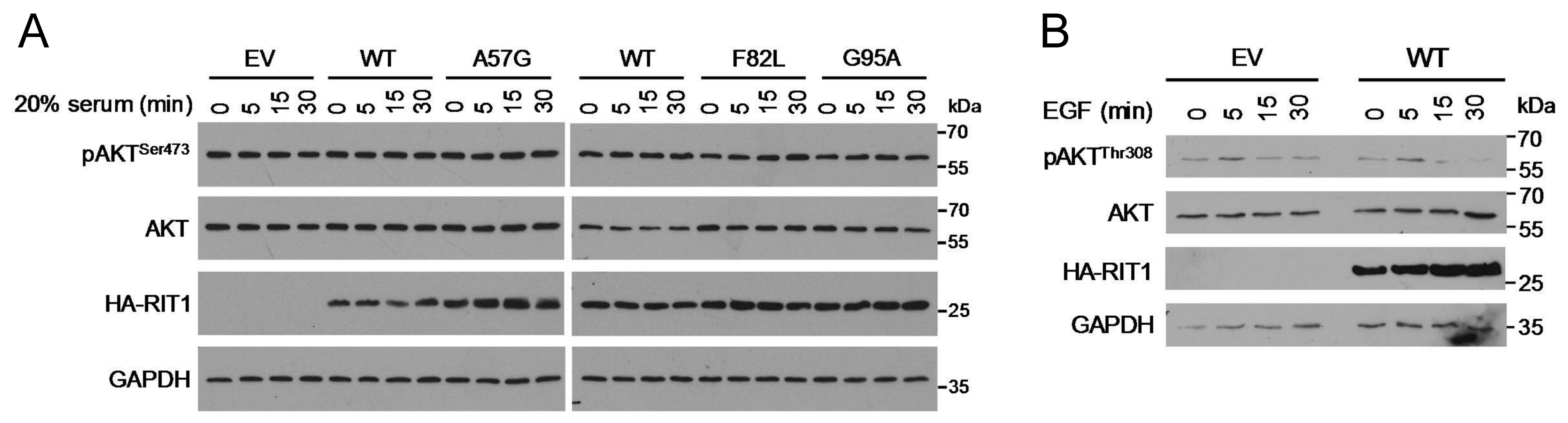

Supplement: S2 Fig — (A) HEK293T cells were transfected with empty vector (EV) and constructs expressing HA-RIT1 wildtype (WT), HA-RIT1 p.A57G, p.F82L or p.G95A as indicated. Cells were cultured under serum-starved condition (0.1% serum; 0 min) and serum-starved condition followed by 5, 15, or 30 min stimulation with 20% serum. Total cell lysates were analyzed by immunoblotting using anti-phospho-AKTSer473 (pAKTSer473) and anti-AKT (AKT) antibodies. Expression of RIT1 protein variants was monitored by immunoblotting using anti-HA antibody, and anti-GAPDH antibody was used to control for equal loading. Data shown are representative of three independent experiments. (B) HEK293T cells were transfected with empty vector (EV) or a construct expressing HA-RIT1 wildtype (WT), cultured under serum-starved condition (0.1% serum; 0 min) and serum-starved condition followed by 5, 15, or 30 min stimulation with 10 ng/ml EGF. Total cell lysates were analyzed by immunoblotting using anti-phospho-AKTThr308 (pAKTThr308) and anti-AKT (AKT) antibodies. Expression of HA-tagged RIT1 protein was monitored by immunoblotting using anti-HA antibody, and anti-GAPDH antibody was used to control for equal loading. Data shown are representative of three independent experiments. (TIF) [file pgen.1007370.s003.tif]

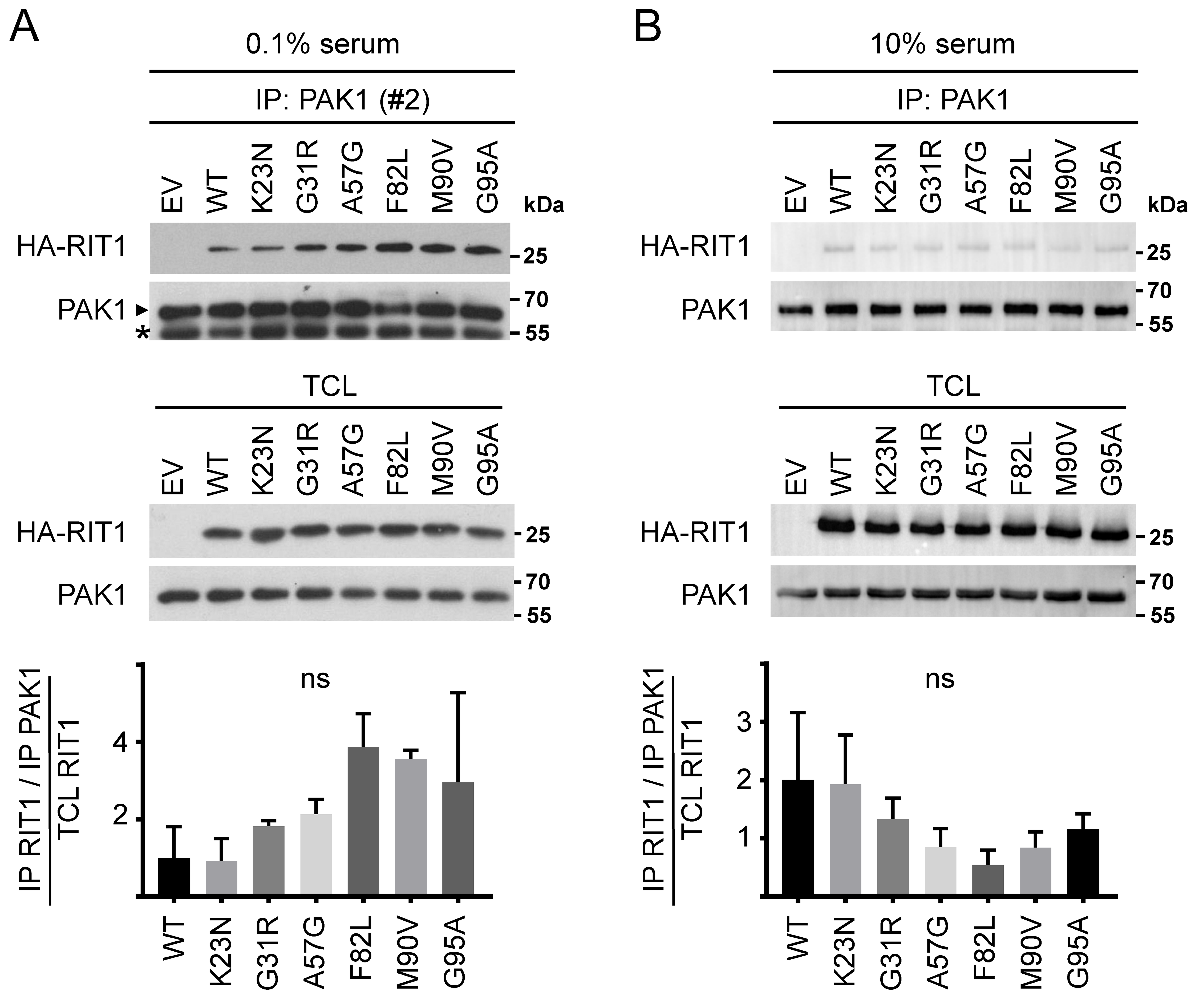

Supplement: S3 Fig — (A and B) HEK293T cells were transfected with empty vector (EV) and RIT1 expression constructs as indicated and cultured under serum deprivation (0.1% serum, A) or basal condition (10% serum, B). Endogenous PAK1 was precipitated with an anti-PAK1 antibody [IP: PAK1 (#2) in (A) from the same extract as shown in Fig 3C; IP: PAK1 (#1) in (B)], and co-precipitated HA-RIT1 was detected using an anti-HA antibody. Enrichment of PAK1 in the precipitates was demonstrated with an anti-PAK1 antibody. The star indicates the heavy chain of the antibody used for precipitation. The amount of HA-RIT1 and PAK1 in total cell lysates (TCL) was monitored by immunoblotting using an anti-HA antibody and an anti-PAK1 antibody, respectively. Data shown are representative of two (A) or three (B) independent experiments. Autoradiographic signals were quantified by scanning densitometry. Levels of co-IPed HA-RIT1 was double-normalized relative to amounts of immunoprecipitated PAK1 and HA-RIT1 in total cell lysates. To conserve the relative variance of the samples, values for RIT1 wildtype and RIT1 mutants were divided by the mean of the wildtype samples [79]. The graphs show the relative amount (arbitrary units) of co-precipitated RIT1 protein variants. The mean of two (A) or three (B) independent experiments ± SD is given, respectively. (A) Unpaired t-tests were used to determine statistical significance. ns, not significant. (B) One-way ANOVA between groups: P < 0.05; ns, not significant. (TIF) [file pgen.1007370.s004.tif]

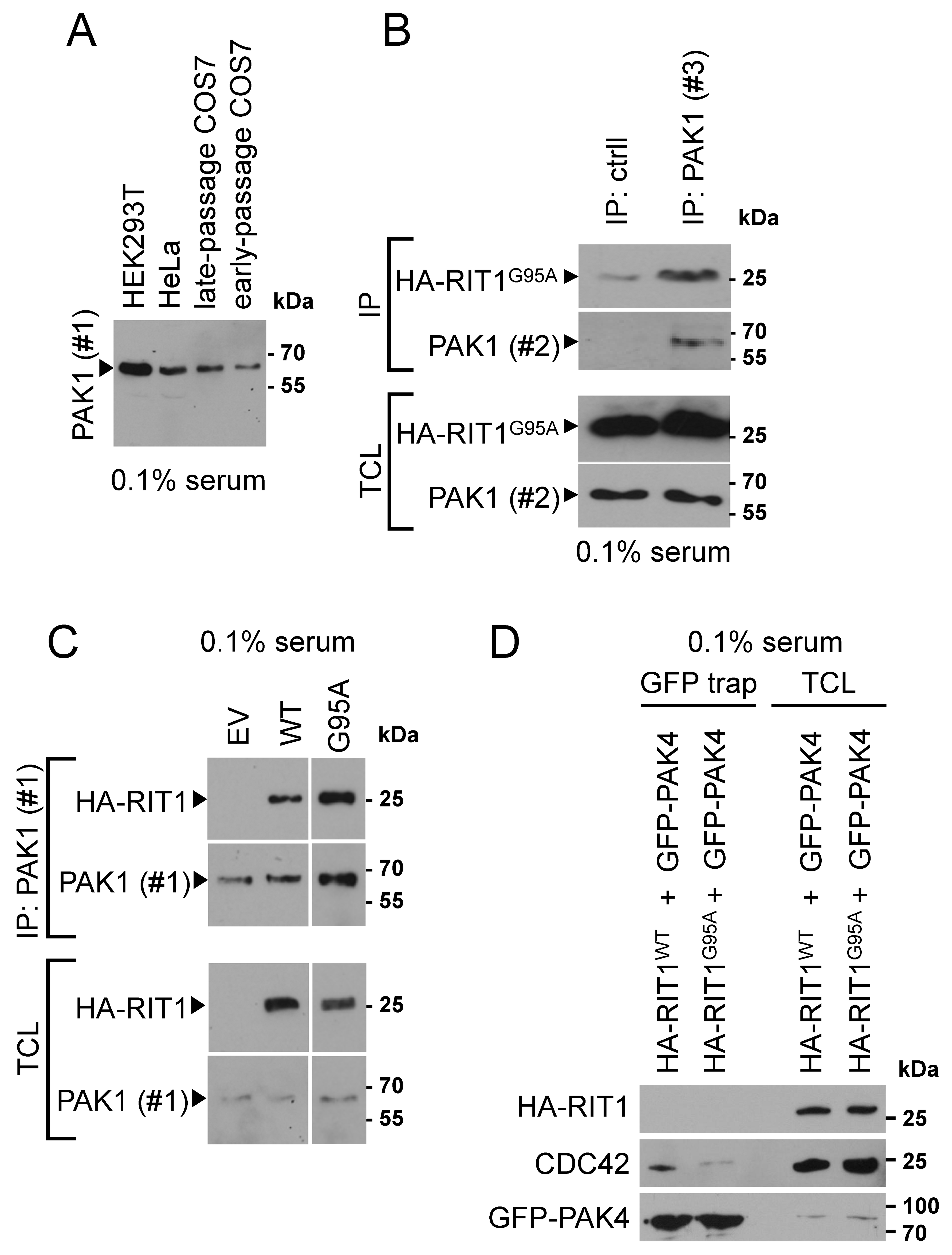

Supplement: S4 Fig — (A) Detection of endogenous PAK1 in serum-starved HEK293T, HeLa and COS7 cells after cell lysis and immunoblotting by using an anti-PAK1 antibody (#1). PAK1 expression is high in HEK293T and weak in COS7 cells. (B) COS7 cells were transfected with HA-RIT1 p.G95A expression construct and cultured under serum deprivation (0.1% serum). Endogenous PAK1 was precipitated with an anti-PAK1 antibody [IP: PAK1 (#3)]. As IP control, an irrelevant isotype-matched antibody (anti-pSMAD2 antibody) was used (IP: ctrl). Enrichment of PAK1 in the precipitates and the amount of endogenous PAK1 in TCL was demonstrated with an anti-PAK1 antibody (#2). Co-precipitated HA-RIT1 and expression of HA-RIT1 in total cell lysates (TCL) was detected by an anti-HA antibody. (C) COS7 cells were transfected with empty vector (EV), RIT1 wildtype or p.G95A expression constructs as indicated and cultured under serum deprivation (0.1% serum). Endogenous PAK1 was precipitated with an anti-PAK1 antibody [IP: PAK1 (#1)]. Enrichment of PAK1 in the precipitates and the amount of endogenous PAK1 in TCL was demonstrated with an anti-PAK1 antibody (#1). Co-precipitated HA-RIT1 and expression of HA-RIT1 in TCL was detected by immunoblotting using an anti-HA antibody. Juxtaposed autoradiographs derive from the same western blotting membrane. (D) HEK293T cells expressing GFP-PAK4 and either HA-tagged RIT1 WT or the HA-RIT1 p.G95A mutant were cultured under serum deprivation (0.1% serum). GFP-PAK4 was precipitated by GFP trap. Enrichment of GFP-PAK4 (GFP trap) is shown by direct comparison of precipitates and TCL. HA-RIT1 was detected by using an anti-HA antibody. As positive IP control, co-precipitated endogenous CDC42 was detected by an anti-CDC42 antibody. Data are representative for three independent experiments. (TIF) [file pgen.1007370.s005.tif]

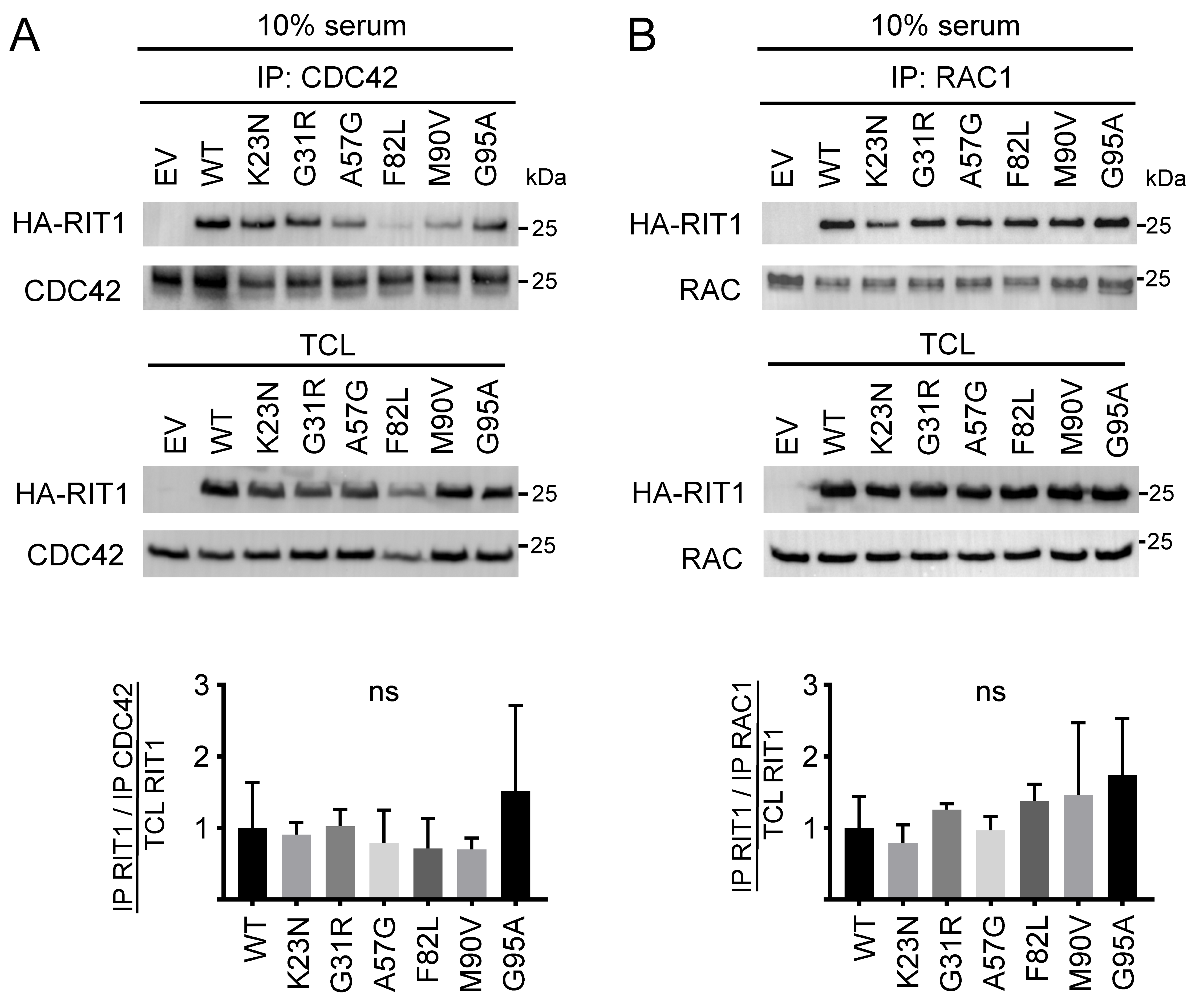

Supplement: S5 Fig — (A and B) HEK293T cells were transfected with empty vector (EV) and RIT1 expression constructs as indicated and cultured under basal condition (10% serum). Endogenous CDC42 and RAC1 were immunoprecipitated with an anti-CDC42 antibody [CDC42 (#3)] (A) and an anti-RAC1 antibody (B), respectively. Co-precipitated HA-RIT1 and HA-RIT in total cell lysates (TCL) was detected by an anti-HA antibody. Enrichment of CDC42 and RAC1 in the precipitates and amount of endogenous CDC42 and RAC1 in TCL was demonstrated with an anti-CDC42 antibody [CDC42 (#4)] (A) and an anti-RAC1/2/3 antibody (B), respectively. Data shown for CDC42 and RAC1 are representative of three (A) and four (B) independent experiments, respectively. Autoradiographic signals were quantified by scanning densitometry. The amount of co-precipitated HA-RIT1 was double-normalized relative to amounts of immunoprecipitated CDC42/RAC1 and HA-RIT1 in total cell lysates. To conserve the relative variance of the samples, values for RIT1 wildtype and RIT1 mutants were divided by the mean of the wildtype samples [79]. The graphs show the relative amount (in arbitrary units) of co-precipitated RIT1 protein variants. The mean of three (A) and four (B) independent experiments ± SD is given. One-way ANOVA between groups: P < 0.05; ns, not significant. (TIF) [file pgen.1007370.s006.tif]

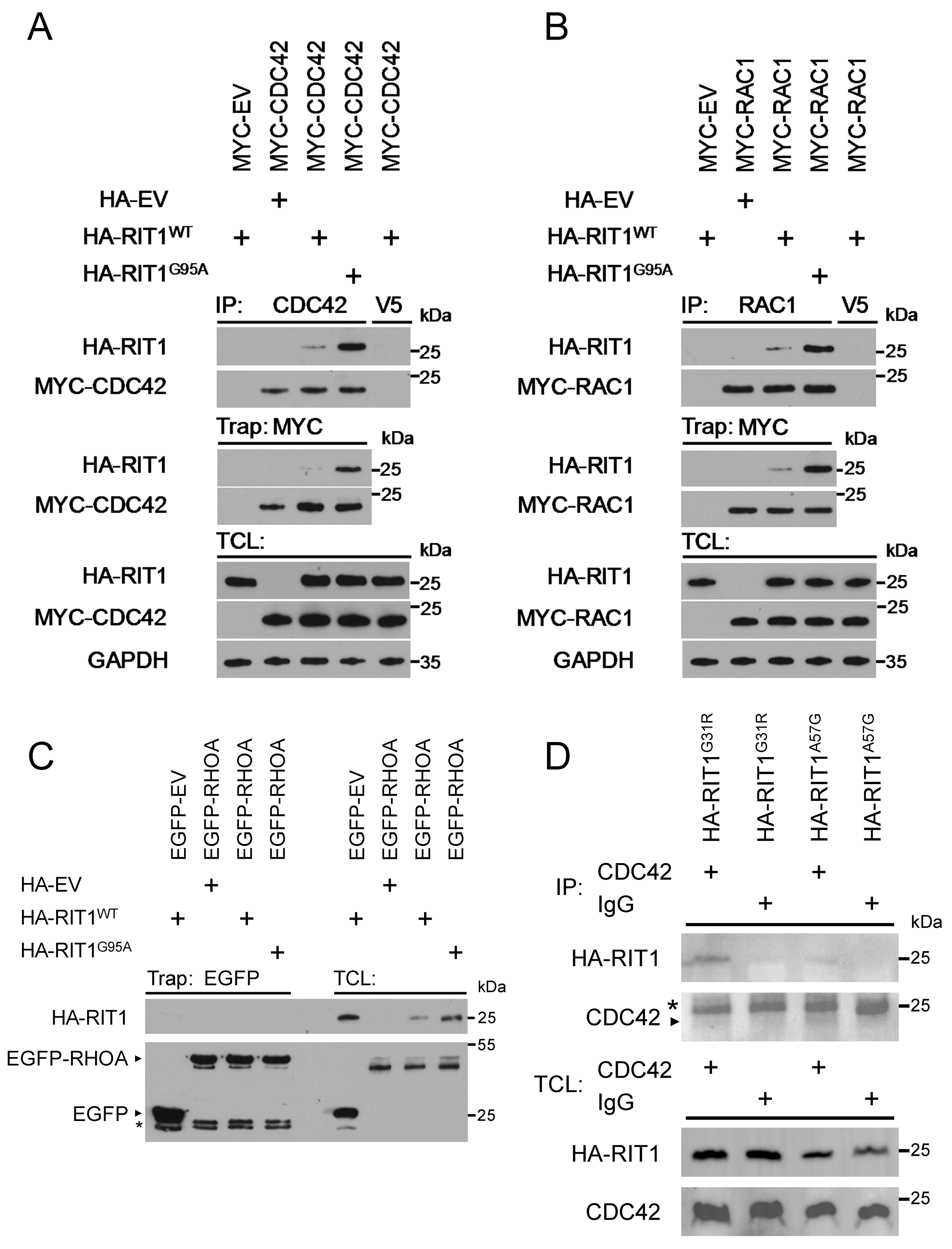

Supplement: S6 Fig — (A and B) HEK293T cells were co-transfected with MYC-empty vector (MYC-EV), MYC-CDC42 (A) or MYC-RAC1 (B) expression construct together with HA-empty vector (HA-EV), RIT1 wildtype (WT) or RIT1 p.G95A expression construct and cultured under serum deprivation (0.1% serum). Lysates were split for precipitation using either anti-MYC coupled beads (Trap MYC in A and B) or primary antibodies against CDC42 (IP CDC42 in A) and RAC1 (IP RAC1 in B). As IP control for primary antibodies an irrelevant isotype-matched antibody against V5 (V5) was used. Both in precipitates (IP, Trap) and total cell lysates (TCL), RIT1 protein was monitored by immunoblotting using an anti-HA antibody, RAC1 and CDC42 were detected by an anti-MYC antibody. Anti-GAPDH antibody was used to control for equal loading. Results are representative for two independent experiments. (C) HEK293T cells were co-transfected with EGFP-empty vector (EGFP-EV) or an EGFP-RHOA expression construct (EGFP-RHOA) together with HA-empty vector (HA-EV), RIT1 wildtype (WT) or RIT1 p.G95A expression construct and cultured under serum deprivation (0.1% serum). Enrichment of EGFP or EGFP-RHOA by EGFP trap (Trap EGFP) is shown by direct comparison of precipitates and total cell lysates (TCL). HA-RIT1 was detected by using an anti-HA antibody and EGFP and EGFP-RHOA by an anti-EGFP antibody. Asterisk indicates the light chain of the antibody used for precipitation. Data are representative for three independent experiments. (D) Endogenous CDC42 of stably transfected Flp-In 293 cells expressing RIT1G31R or RIT1A57G was immoprecipitated with an anti-CDC42 antibody [IP: CDC42 (#5)]. Endogenous CDC42 protein in total cell lysates (TCL) and precipitates was detected with an anti-CDC42 antibody [WB: CDC42 (#4)]. Co-precipitated HA-RIT1 and HA-RIT1 in TCL was detected by an anti-HA antibody. As a control, an isotype-matched non-specific anti-IgG rabbit antibody (IgG) was used for immunoprecipitation. The asterisk indicates the light chai [file pgen.1007370.s007.tif]

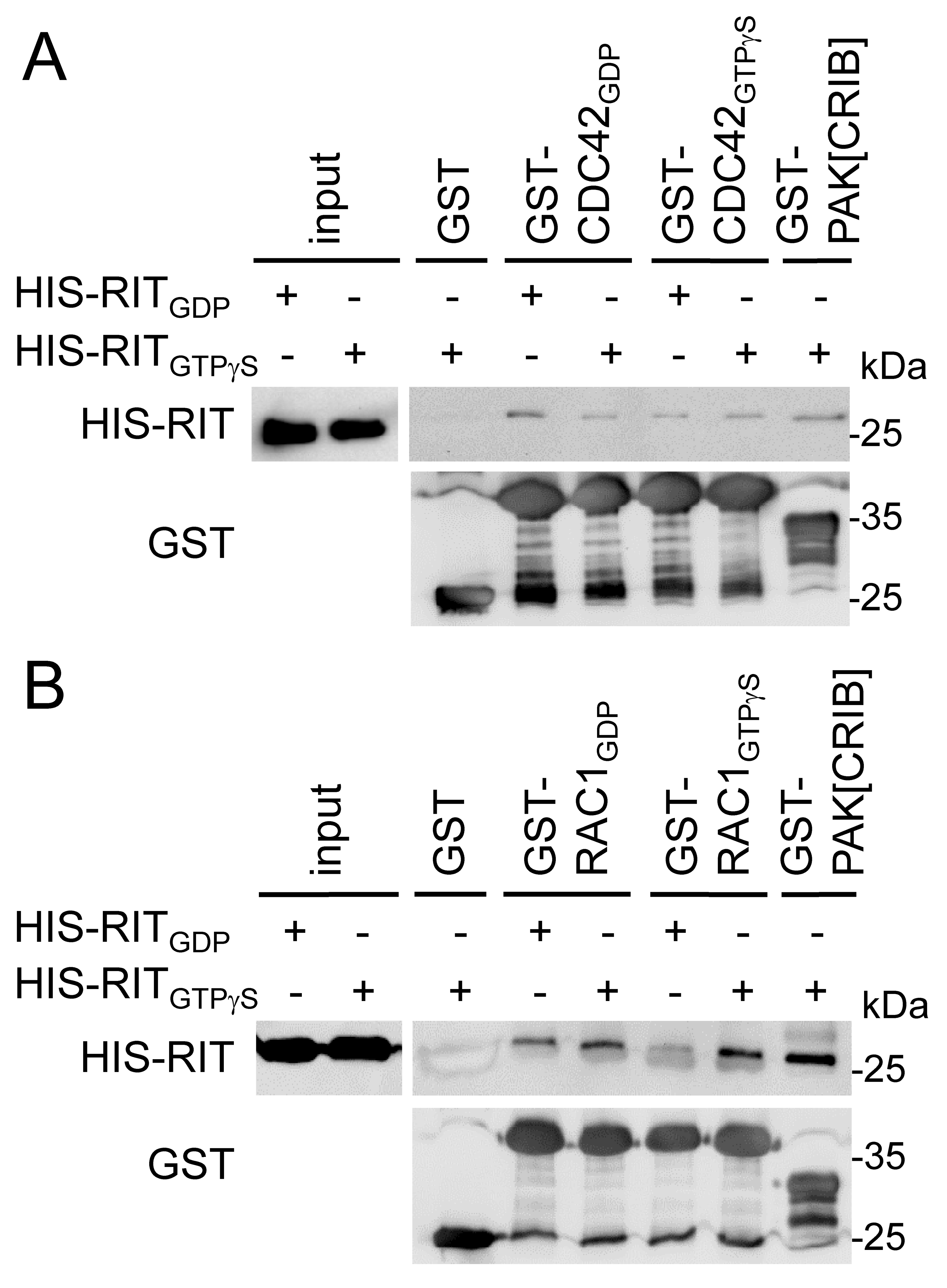

Supplement: S7 Fig — Recombinant His-tagged RIT1 wildtype (0.5 μM) was loaded with GDP or non-hydrolyzable GTPγS as indicated and incubated with 1 μM GST-CDC42 (loaded with GDP or GTPγS) (A) or GST-RAC1 (loaded with GDP or GTPγS) (B). Recombinant GST (1 μM) and GST-PAK[CRIB] (1 μM) were used as negative and positive control, respectively. Glutathione agarose-coupled GST fusion proteins were precipitated and samples were analyzed by immunoblotting using an anti-His antibody (precipitates and input) and an anti-GST antibody (precipitates). Data shown are representative of three independent experiments. (TIF) [file pgen.1007370.s008.tif]

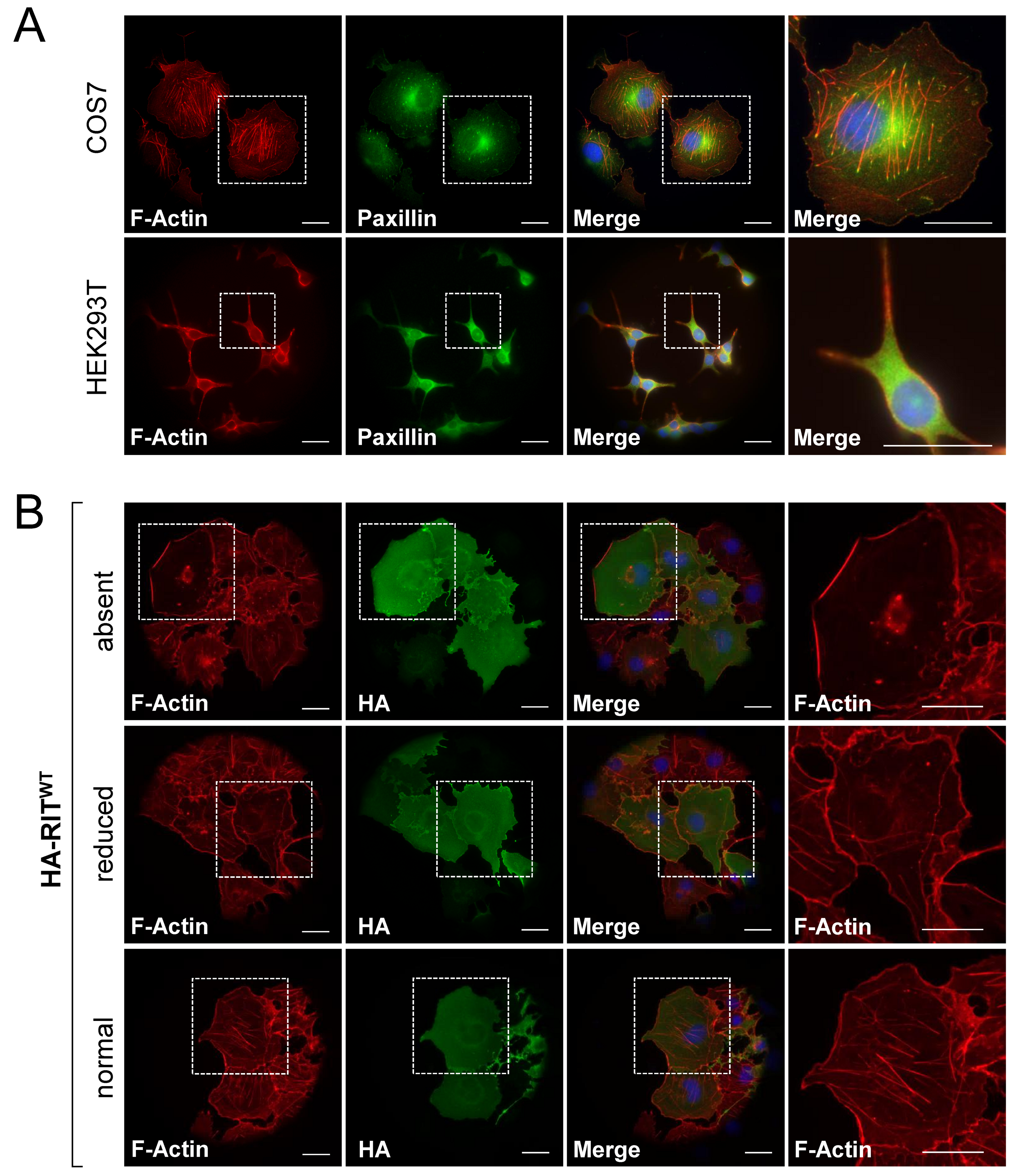

Supplement: S8 Fig — (A) COS7 and HEK293T cells were plated on collagen-coated glass slides and kept under serum starvation overnight. F-actin was visualized using Texas Red-X Phalloidin. Paxillin was stained using mouse anti-paxillin antibody and Alexa Fluor488-conjugated anti-mouse antibody. Nuclear DNA was labeled by DAPI. White boxes indicate magnified parts of specimen shown on the very right-hand side. Scale bar, 10 μm. (B) COS7 cells were plated onto collagen-coated coverslips, transiently transfected with the HA-RITWT construct and serum-starved overnight. Cells were stained with mouse anti-HA-antibody followed by anti-mouse Alexa Fluor 488-conjugated antibody, Texas Red-X Phalloidin for actin distribution and DAPI for nucleus staining. White boxes indicate magnified parts of specimen shown on the right-hand side. Scale bars, 10 μm. Exemplary images depicting cells with normal, reduced or absent actin stress fibers are shown. (TIF) [file pgen.1007370.s009.tif]

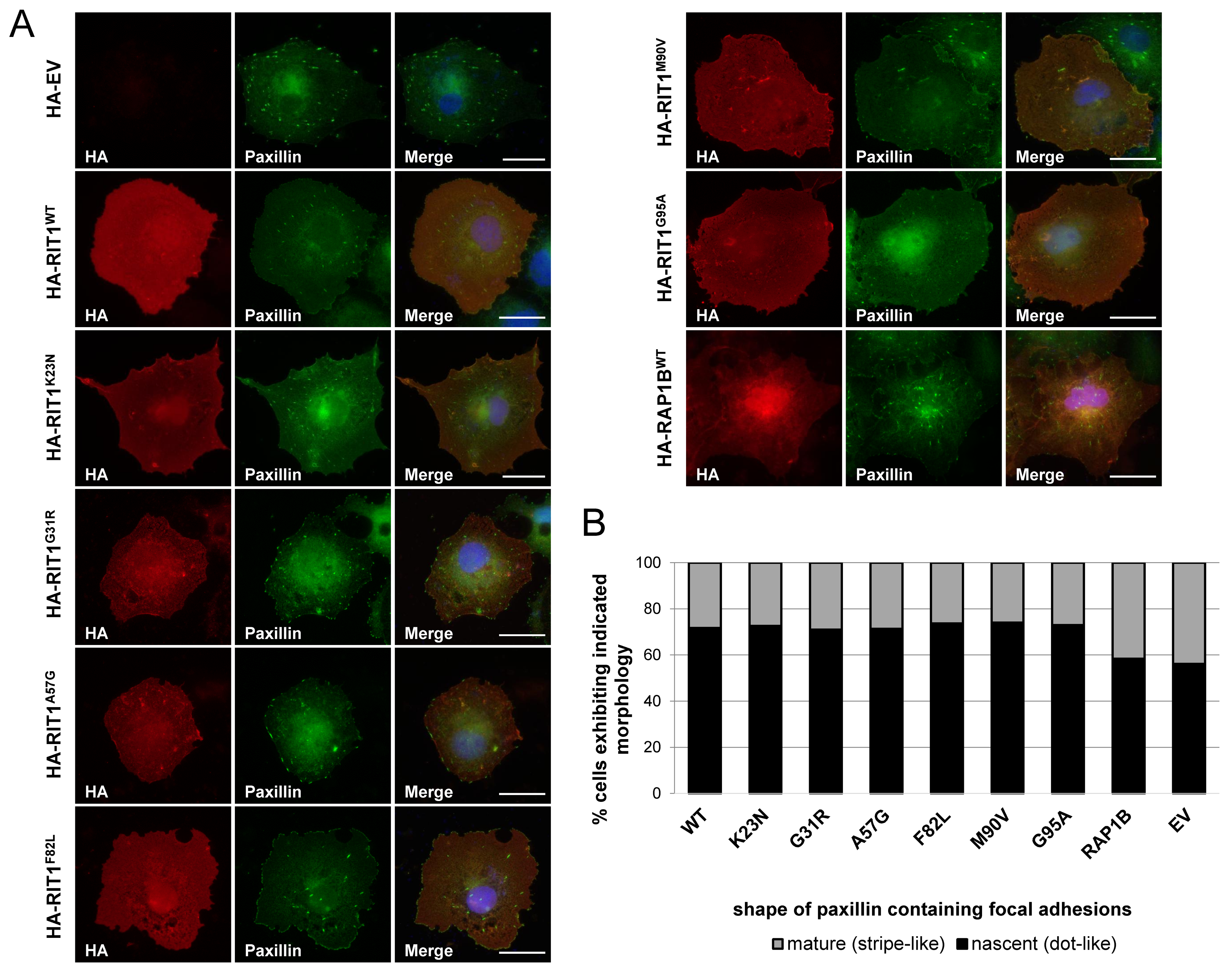

Supplement: S9 Fig — (A) COS7 cells were plated on collagen-coated glass slides, transiently transfected with the indicated construct and kept under serum starvation overnight. HA-tagged RIT1 was stained by rabbit anti-HA antibody followed by anti-rabbit Alexa Fluor546-conjugated antibody. Paxillin was visualized using mouse anti-paxillin antibody and Alexa Fluor488-conjugated anti-mouse antibody. Nuclear DNA was labeled by DAPI. Scale bar, 10 μm. (B) The number of nascent (dot-like) and mature (stripe-like) paxillin-positive structures per cell was determined in at least 30 cells of each dataset. (TIF) [file pgen.1007370.s010.tif]

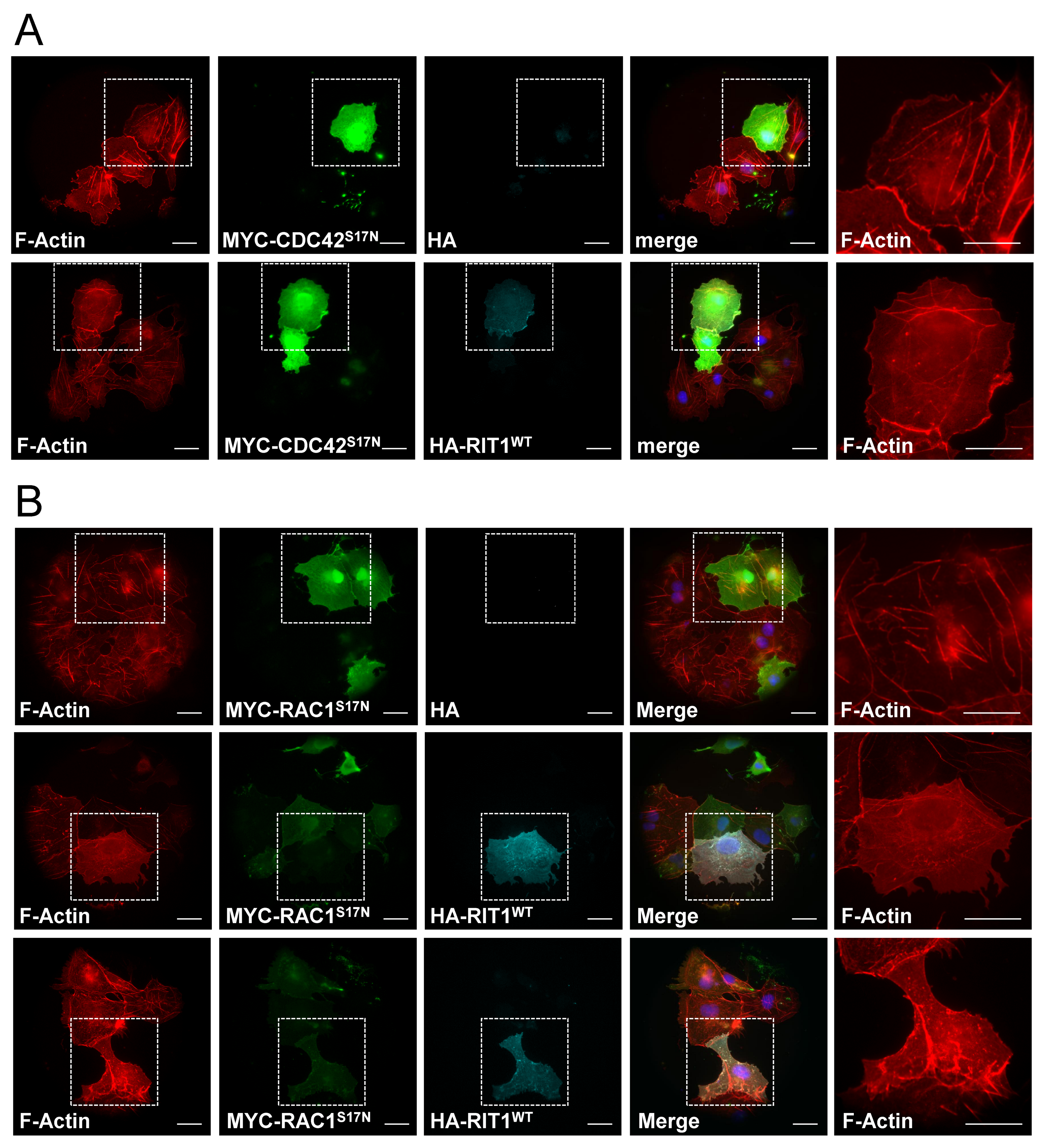

Supplement: S10 Fig — COS7 cells were plated on collagen-coated glass slides, transiently transfected with the indicated constructs and serum-starved overnight. MYC-tagged CDC42S17N (A) and RAC1S17N (B) were stained by mouse anti-MYC antibody followed by anti-mouse Alexa Fluor488-conjugated antibody. HA-tagged RIT1 was visualized using rabbit anti-HA antibody and anti-rabbit Alexa Fluor647-conjugated antibody. F-actin was stained by using Texas Red-X Phalloidin, and nuclear DNA was labeled by DAPI. The label “HA” indicates cell(s) not transfected with the RIT1 wildtype construct. For co-expression of RAC1S17N and RIT1 wildtype two separate microscopic data series are shown (last two rows in B). White boxes indicate magnified parts of specimen shown on the very right-hand side. Scale bar, 10 μm. (TIF) [file pgen.1007370.s011.tif]

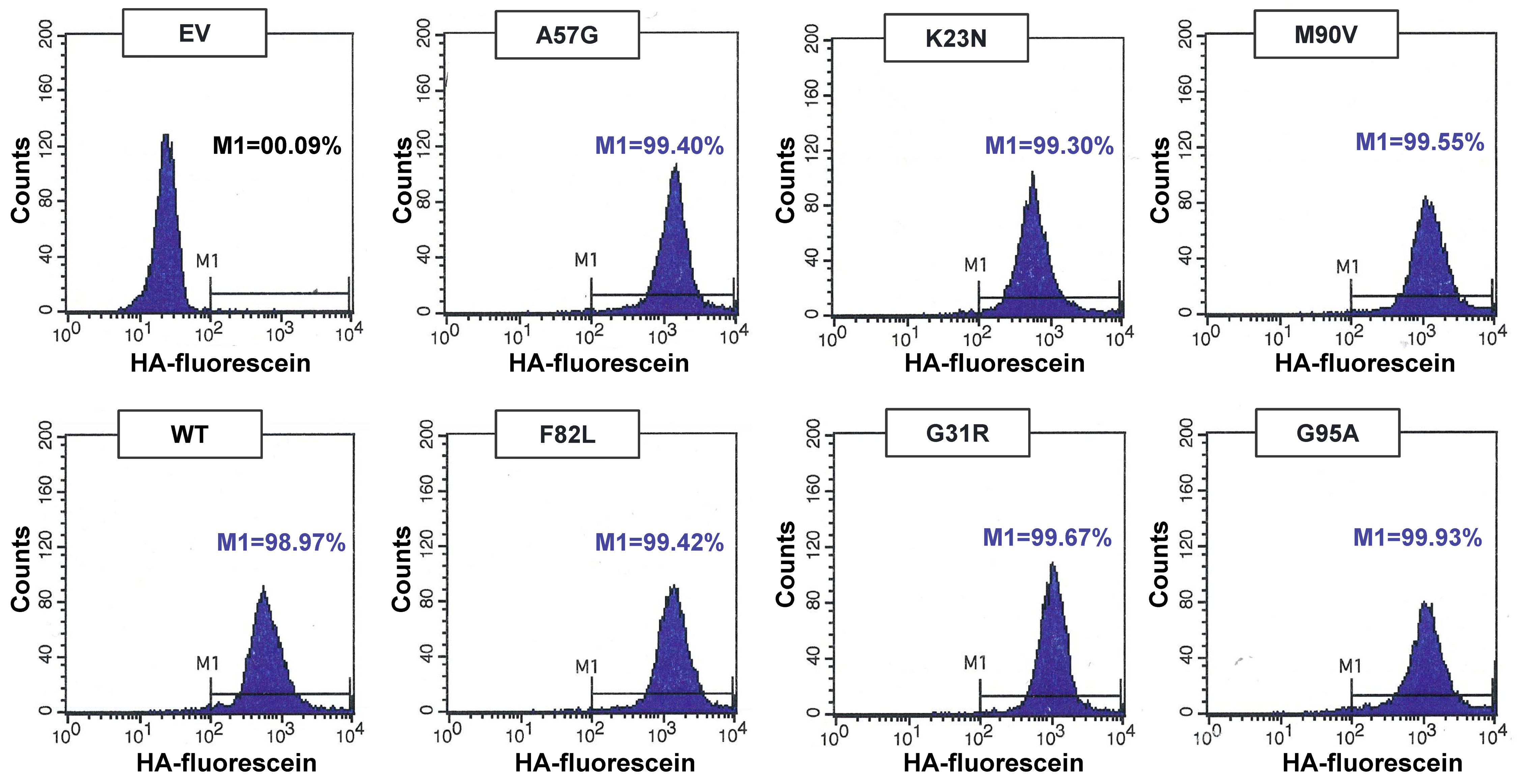

Supplement: S11 Fig — HEK293T cells were transiently transfected with cDNA constructs expressing the indicated HA-tagged RIT1 protein variants and serum-starved overnight. Cells were stained with anti-HA-fluorescein antibody and counted by flow cytometry. Percentage of cells positive for HA-fluorescein staining is indicated for each construct. (TIF) [file pgen.1007370.s012.tif]
